# Atomistic Modeling of Scattering Curves for Human IgG1/4 Reveals New Structure-Function Insights

**DOI:** 10.1016/j.bpj.2019.10.024

**Published:** 2019-10-24

**Authors:** David W. Wright, Emma L.K. Elliston, Gar Kay Hui, Stephen J. Perkins

**Affiliations:** 1 Department of Structural and Molecular Biology, Division of Biosciences, University College London, London, United Kingdom

## Abstract

Small angle x-ray and neutron scattering are techniques that give solution structures for large macromolecules. The creation of physically realistic atomistic models from known high-resolution structures to determine joint x-ray and neutron scattering best-fit structures offers a, to our knowledge, new method that significantly enhances the utility of scattering. To validate this approach, we determined scattering curves for two human antibody subclasses, immunoglobulin G (IgG) 1 and IgG4, on five different x-ray and neutron instruments to show that these were reproducible, then we modeled these by Monte Carlo simulations. The two antibodies have different hinge lengths that connect their antigen-binding Fab and effector-binding Fc regions. Starting from 231,492 and 190,437 acceptable conformations for IgG1 and IgG4, respectively, joint x-ray and neutron scattering curve fits gave low goodness-of-fit *R* factors for 28 IgG1 and 2748 IgG4 structures that satisfied the disulphide connectivity in their hinges. These joint best-fit structures showed that the best-fit IgG1 models had a greater separation between the centers of their Fab regions than those for IgG4, in agreement with their hinge lengths of 15 and 12 residues, respectively. The resulting asymmetric IgG1 solution structures resembled its crystal structure. Both symmetric and asymmetric solution structures were determined for IgG4. Docking simulations with our best-fit IgG4 structures showed greater steric clashes with its receptor to explain its weaker Fc*γ*RI receptor binding compared to our best-fit IgG1 structures with fewer clashes and stronger receptor binding. Compared to earlier approaches for fitting molecular antibody structures by solution scattering, we conclude that this joint fit approach based on x-ray and neutron scattering data, combined with Monte Carlo simulations, significantly improved our understanding of antibody solution structures. The atomistic nature of the output extended our understanding of known functional differences in Fc receptor binding between IgG1 and IgG4.

## Significance

Atomistic solution structural studies from joint x-ray and neutron scattering curve fits, when combined with molecular dynamics and Monte Carlo simulations, provide a, to our knowledge, new means of determining structures. Here, this method was evaluated in detail using multiple x-ray and neutron data sets for human immunoglobulin G (IgG) 1 and IgG4 antibody subclasses. Single small families of best-fit structures were determined for both subclasses after starting from a large library of simulated structures. These structures explained the different binding modes of IgG1 and IgG4 to two different Fc receptors at a molecular level, thus illustrating the value of this new method to study antibody function. We discuss the applicability of this joint x-ray and neutron approach combined with structural simulations to other similar systems.

## Introduction

**Figure 1:**
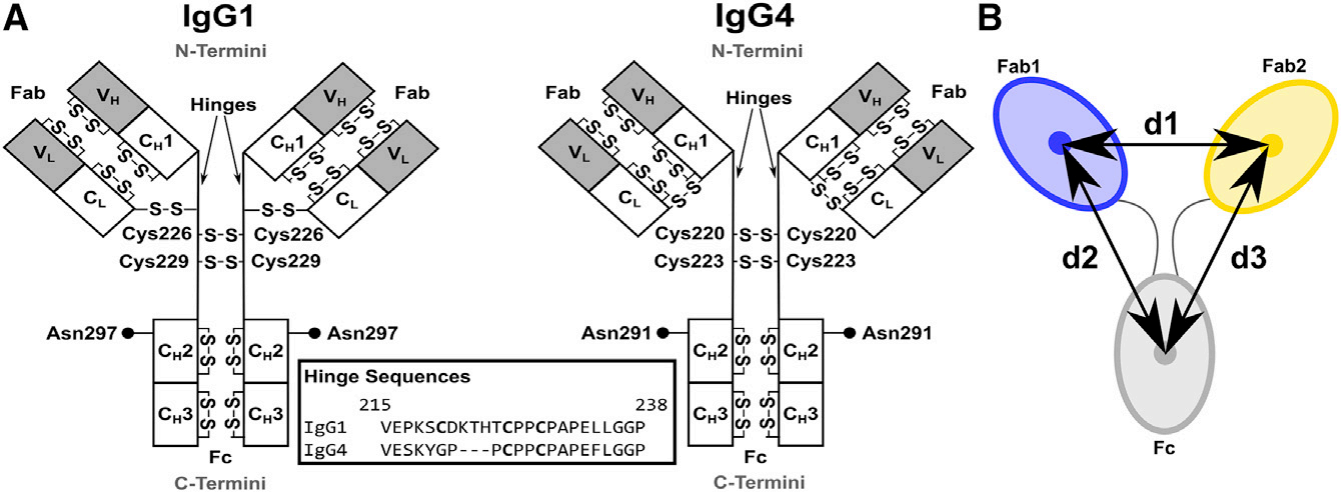
Domain structures of human IgG1 and IgG4. (*A*) Each heavy chain of IgG1 and IgG4 is formed from the variable and constant V_H_, C_H_1, C_H_2, and C_H_3 domains. Each light chain is formed from the V_L_ and C_L_ domains. The hinge sequences in the inset are shown in EU numbering. Two interchain disulphide bridges connect the two heavy chains. Two conserved N-glycosylation sites in the Fc region are at Asn297 (●). (*B*) A cartoon of the Fab and Fc regions. The distance between the centers of mass of the two Fab regions was denoted as d1. Those between the two Fab and Fc regions were denoted as d2 and d3. The antibody is shown arbitrarily as a two-fold symmetric structure with d2 = d3. In general, d2 and d3 are unequal. In the text, the smaller of the two values is denoted as min(d2, d3), and the larger of the two is denoted as max(d2, d3).

Antibodies are glycoproteins that protect the host by identifying and neutralizing pathogens. They mediate highly specific antigen binding to a specific epitope through their two Fab regions, followed by their effector binding to other components of the immune system through its Fc region (Fig. 1). Immunoglobulin G (IgG) is the most abundant of the five human antibody classes. In the four IgG subclasses IgG1–IgG4, IgG1 is the most prevalent in serum, and IgG4 is the least. In IgG, the heavy and light chains are paired to form two Fab regions that are joined by two polypeptide hinges linked by interchain disulphide bonds to the Fc region that is formed from two heavy chains (Fig. 1). The variable domains (V_H_ and V_L_) mediate antigen binding, whereas the Fc constant domains (C_H_2 and C_H_3) perform effector functions (1,2). The Fc region also possesses an N-linked glycosylation site at Asn297. IgG1–IgG4 exhibit over 90% sequence identity, differing primarily in their hinges and upper C_H_2 domains (3). The IgG1 and IgG4 hinges are of lengths 23 and 20 residues, respectively, whereas that of IgG2 is similar and that of IgG3 is much longer. The IgG1 and IgG4 hinges contain two interchain disulphide bonds, although these in IgG4 can interconnect differently to form hinge isomers (4). Consequently, IgG1 and IgG4 exhibit different effector functions in terms of receptor and complement binding from conformational variations in their hinge region (Fig. 1 *A*). Flexibility in the hinge is also relevant for function, this being exemplified in the stochastic walking of antibodies on repetitive antigenic epitopes such as on viral surfaces (5).

Antibodies are increasingly important in therapeutic applications (6,7). Over 294 monoclonal-antibody-based drugs have been approved or are in review with worldwide revenues of over $50 billion (8,9). Knowledge of the relationships between antibody sequence, structure, and function in physiological and manufacturing conditions is required for antibody engineering and therapeutics. This knowledge is limited by the very few crystal structures that are known for full-length antibodies, let alone their complexes with receptors; thus, there is great interest in determining their molecular structures, especially in solution. Three-dimensional antibody solution structures can be investigated using small angle x-ray scattering (SAXS) (10, 11, 12), small angle neutron scattering (SANS) (13), or both SAXS and SANS in conjunction with analytical ultracentrifugation (14, 15, 16). In SAXS or SANS, the scattering intensities result from the density contrast difference between the antibody and the solvent (17). SAXS and SANS monitor different aspects of the solution structure. For example, the tightly bound first hydration shell surrounding the protein is visible in SAXS but much less so in SANS if the protein is studied in heavy water buffer (18, 19, 20). Traditionally, SAXS and SANS studies are low-resolution structural methods that are unable to derive reliable unique structures. The realization that molecular structures can be used to fit scattering curves and that comparatively few molecular structures result in good scattering fits gives rise to a powerful approach for determining molecular structures. In this study, atomistic modeling fits of the scattering curves based on constraints from known crystal structures and protein sequences leads to the determination of molecular structures for the antibody solution structures. This is advantageous compared to the use of small beads (or spheres) or surface envelopes to fit the scattering curves because the resulting atomistic structures lead to clarifications of the molecular basis of antibody function in antigen or receptor binding. In our earlier antibody modeling, the SCT/SCTPL modeling package utilized separate crystal structures for the Fab and Fc regions that were combined with randomized hinge structures to create intact antibody structures that were fitted against SAXS-SANS data. The SCT/SCTPL package resulted in best-fit molecular structures, but it was limited by the creation of redundant models, sometimes with sterically overlapping regions, and was computationally expensive. SCT/SCTPL resulted in 14 full antibody solution structures available in Protein Data Bank (PDB) format (21), including human IgG1 and IgG4 (15,16) and rabbit IgG (14).

In the CCP-SAS project, SCT/SCTPL has been superseded by the SASSIE workflow on high-performance computing hardware to rapidly create larger numbers of protein conformations that are now physically correct (22,23). SCT/SCTPL and SASSIE represent two examples of the growth of atomistic modeling approaches to interpret scattering data (reviewed in (23)). First, in SASSIE, a full energy-minimized starting model was created. Then, Monte Carlo variations of the polypeptide main chain *θ* and *ψ* dihedral angles rapidly generated further models in which models with poor steric overlaps were discarded at the point of generation. These were fitted against scattering data. SASSIE was used to determine a solution structure for human monoclonal IgG2 from SANS data (13). Here, to validate this method in greater detail, SASSIE was used to remodel our previous joint SAXS and SANS data sets for human monoclonal IgG1 and IgG4 (15,16) alongside new joint IgG1 and IgG4 scattering data from three other instruments to test their experimental reproducibility. Initial structures for full-length IgG1 and IgG4 were thus created using molecular dynamics. Monte Carlo simulations rapidly generated ∼700,000 physically realistic IgG1 and IgG4 trial models to explore the physically allowed conformational space around the hinge region. Joint filters based on disulphide bridge constraints and consistency with joint SAXS and SANS fits resulted in a limited number of new atomistic solution structures that reflected the different hinge lengths of IgG1 and IgG4. The resulting models confirmed in greater detail and rigor our previous SCT/SCTPL analyses of IgG1 and IgG4 (15,16). Docking studies of these best-fit full-length IgG structures with their receptors provided, to our knowledge, new insight into the different functional roles of IgG1 and IgG4; therefore, the use of SASSIE is able to enhance our understanding of antibody-receptor-binding function.

## Materials and Methods

### Source of IgG1 and IgG4 antibodies

The monoclonal IgG1 6a and IgG1 19a antibodies were used here as previously described (16). The monoclonal IgG4 B72.3 antibody in its Ser222 and Pro222 forms was also used as previously described, with the Ser222 form corresponding to the wild-type hinge and the Pro222 form having a stabilized IgG4 hinge structure that prevents Fab-arm exchange (15). For new data collection, both IgG1 and IgG4 antibodies were purified by size-exclusion chromatography using a Superose 6 10/300 column (GE Healthcare, Chicago, IL) to remove nonspecific aggregates immediately before the SAXS and SANS measurements. For x-ray work, all four antibodies were measured in phosphate-buffered saline (PBS)-137 (137 mM NaCl, 8.1 mM Na_2_HPO_4_, 2.7 mM KCl, 1.5 mM KH_2_PO_4_ (pH 7.4)), and replacing 137 mM NaCl with 50 mM NaCl or 250 mM NaCl in buffers termed PBS-50 and PBS-250. For neutron work, the light water in PBS-137 was replaced by 100% heavy water by extensive dialysis into 100% heavy water immediately before SANS experiments. By this, the H atoms in the hydration shell are replaced by D atoms, together with 90% of the protein-exchangeable H-atom content (18). Additional data collection utilized Hepes-137 buffer (10 mM Hepes, 137 mM NaCl, and 2 mM CaCl_2_ (pH 7.4)).

### SAXS and SANS data for IgG1 and IgG4

Our previous SAXS data for IgG1 6a and 19a and IgG4 B72.3 in PBS-50, PBS-137, and PBS-250 buffers (15,16) were recorded in 16-bunch mode on instrument ID02 at the European Synchrotron Radiation Facility, Grenoble, France (24). This used a detector with a resolution of 512 × 512 pixels in sets of 10 time frames with exposure times of 0.1 or 0.2 s each. New SAXS data (experimental ID: MX1801) were acquired using the BioSAXS robot on instrument BM29 at the European Synchrotron Radiation Facility (25,26). Data collection utilized a CMOS hybrid pixel Pilatus 1M detector (Dectris, Baden, Switzerland) with a resolution of 981 × 1043 pixels (pixel size of 172 × 172 *μ*m). Overall, IgG1 6a and IgG1 19a were studied at eight different concentrations between 0.04 and 0.33 mg/mL and 0.12–0.96 mg/mL, respectively. Sample volumes of 50 *μ*L were used in the BioSAXS automatic sample changer. Each sample was moved continuously in the capillary during beam exposure to reduce radiation damage. 10 time frames, each of duration 0.1 s, were acquired, alongside online checks that confirmed the absence of radiation damage during data acquisition. After this, the 10 frames were averaged. The BsxCUBE GUI interface was used for control of the automatic sample changer, and the sample settings were loaded from the ISPyB interface (25,27,28).

Our previous SANS data for IgG1 6a and IgG4 B72.3 were obtained in PBS-137 in 100% ^2^H_2_O at 6, 20, and 37°C on instrument SANS2d at the ISIS pulsed neutron source, Rutherford Appleton Laboratory, Didcot, UK (29). New SANS data for IgG1 6a, IgG1 19a, and IgG4 B72.3 in the same buffer were obtained on instruments D11 (DOI: 10.5291/ILL-DATA.8-03-846) and D22 (DOI: 10.5291/ILL-DATA.8-03-832) at the Institut Laue-Langevin, Grenoble, France (30). For D11, samples were measured at sample-to-detector distances of 1.2 and 8 m, with 5.5 and 8 m collimation and a wavelength *λ* of 0.60 nm. For D22, the sample-to-detector and collimation distances were 5.6 m with *λ* of 0.60 nm. All samples were measured in rectangular Hellma cells with 2 mm thickness in a thermostatted sample rack set at 20°C. Data for IgG1 6a were collected at four concentrations of 0.5–3.91 mg/mL on D11 in Hepes-137 in 100% ^2^H_2_O and at 0.34 mg/mL on D22 in PBS-137 in 100% ^2^H_2_O. Data for IgG1 19a were collected at five concentrations of 0.5–2.83 mg/mL on D11 in Hepes-137 in 100% ^2^H_2_O and at three concentrations of 0.32–0.96 mg/mL on D22 in PBS-137 in 100% ^2^H_2_O. Data for IgG4 B72.3 were collected on D22 at five concentrations of 0.5–4 mg/mL in PBS-50, 0.5–4 mg/mL in PBS-137, and 0.4–3.2 mg/mL in PBS-250, all in 100% ^2^H_2_O.

### Scattering curve analyses of IgG

For macromolecules measured in high solute-solvent contrasts, the radius of gyration *R*_*g*_ is a measure of structural elongation if it is assumed that the internal inhomogeneity of scattering densities has no effect. This is well-approximated by x-ray measurements in physiological salt buffers or by neutron measurements in 100% ^2^H_2_O. Guinier analyses of the scattering curve *I*(*Q*) at low scattering vectors, *Q* (where *Q* = 4*π*sin*θ*/*λ*; 2*θ* is the scattering angle), give the *R*_*g*_ and the forward scattering at zero angle *I*(0) (31):

(1) $$\ln\;I\left(Q\right)=\ln\;I\left(0\right)-\frac{R_{g}^{2}Q^{2}}{3}.$$

This expression is valid in a *Q*, *R*_*g*_ range up to 1.5. If the structure is elongated, and represents shapes similar to long rods, the mean radius of gyration of the cross-sectional structure *R*_*xs*_ is obtained from the convolution of the scattering curve with a cross-sectional factor represented by *Q* (31,32):

(2) $$\ln\left[I\left(Q\right)\times Q\right]={\left[I\left(Q\right)\times Q\right]}_{Q\to 0}-\frac{R_{xs}^{2}Q^{2}}{2}.$$

The radius of gyration of the cross section is a monitor of the mean width of an elongated structure. The cross-sectional plot for antibodies exhibits two distinct linear regions, a steeper innermost one and a flatter outermost one (32). The two analyses are denoted as *R*_*xs*1_ and *R*_*xs*2_, respectively. The *R*_*xs*1_ parameter monitors the mean width of the full antibody structure, whereas the *R*_*xs*2_ parameter monitors the mean width of each of the individual Fab and Fc structures (Fig. 1). The *R*_*g*_ and *R*_*xs*_ analyses were performed using the SCT package (21). For IgG1, the *Q* ranges for *R*_*g*_, *R*_*xs*1_, and *R*_*xs*2_ that gave linear fits were 0.15–0.28, 0.31–0.47, and 0.65–1.04 nm^−1^, respectively (16). For IgG4, the same *Q* ranges were used for *R*_*g*_ and *R*_*xs*2_, whereas *R*_*xs*1_ was calculated from an adjusted *Q* range of 0.31–0.51 nm^−1^ for reason of its slightly different shape (15). Indirect Fourier transformation of the scattering data *I*(*Q*) in reciprocal space into the distance distribution function *P*(*r*) in real space was carried out using the program ScÅtter (http://www.bioisis.net/users/sign_in):

(3) $$P\left(r\right)=\frac{1}{2\pi^{2\;}}\;\int_{0}^{\infty }I\left(Q\right)\times Q\;r\;\sin\left(Qr\right)dQ.$$

*P*(*r*) corresponds to the distribution of distances *r* between all volume elements. This yields the maximum dimension of the macromolecule *L* and its most commonly occurring distance vector *M* in real space, as well as an alternative calculation of the *R*_*g*_ value.

### Generation of initial IgG structural models

Atomistic scattering modeling compares theoretical scattering curves calculated from protein crystal structural models with the experimental scattering curves. For this, the antibody amino-acid residues were numbered using standard EU numbering for IgG (33,34).

The previous SCT/SCTPL modeling of IgG1 (16) utilized the crystal structure of full-length human IgG1 b12 (PDB: 1HZH) (35) to create 20,000 symmetric and asymmetric randomized full-length models of IgG1. For these, either the seven-residue upper hinge ^220^CDKTHTC^226^ with Cys^220^ and Cys^226^ acting as tethers was randomized to make asymmetric IgG1 structures or the 19-residue upper, middle, and lower hinge ^220^CDKTHTCPPCPAPELLGGP^238^ was randomized to make both symmetric and asymmetric IgG1 structures. Sequence differences between IgG1 b12, IgG1 6a, or IgG1 19a were disregarded. For the previous SCT/SCTPL modeling of IgG4 (15), the starting model was constructed from crystal structures for Fab B72.3 and human IgG1 b12 (PDB: 1BBJ and 1HZH) (36,37). The asymmetric IgG4 models considered only the upper hinge ^212^VESKYGPPC^220^ with Val^212^ and Cys^220^ acting as tethers to create 10,000 randomized IgG4 models. Symmetric IgG4 models considered the upper, middle, and lower hinges as a 21-residue peptide ^212^VESKYGPPCPSCPAPEFLGGP^232^ to create another 10,000 IgG4 models.

For our SASSIE modeling, the starting IgG1 model also employed the crystal structure of full-length human IgG1 b12 (PDB: 1HZH) (35). 13 residues were not present in this 1HZH structure, specifically ^132^SKSTSGG^138^ in one Fab C_H_1 domain, ^223^THT^225^ in its associated core hinge, and ^445^PGL^447^ at one of the two C-termini in the Fc C_H_3 domain. The missing Fab region and hinge residues were reconstructed by duplicating the coordinates of residues 1–299 with those from the other complete heavy chain, superimposed on residue 229 of the starting heavy chain. The missing three C-terminal residues were modeled with backbone *φ* and *ψ* angles of 10° using the PyMOL build_seq script in the PyMOL Script Repository, Queen’s University, Ontario, Canada (Schrödinger). All disulphide bonds were retained. For this complete IgG1 starting structure, force field parameterizations were generated and hydrogen atoms added using the glycan reader component of CHARMM-GUI (37,38) and the CHARMM36 force field (39, 40, 41, 42). This IgG1 structure was energy-minimized for 1000 steps using the conjugate gradient method implemented in NAMD2 (43). Of the two disulphide bonds at Cys226 and Cys229 (Fig. 1 *A*), only that at Cys226-Cys226 was present in the IgG1 b12 crystal structure. The Cys226-Cys226 conformation was retained in the initial model, with the force field parameterizations being varied to incorporate or exclude it as required below. Sequence differences between IgG1 b12, IgG1 6a, or IgG1 19a were again disregarded. Two biantennary Gal_2_.GlcNAc_2_.Man_3_.GlcNAc_2_ glycans at Asn297 were retained (Fig. 1 *A*). The x-ray and neutron scattering length density of the glycans is slightly higher than that of the protein; thus, the glycan and protein components were indistinguishable by scattering (18).

For our SASSIE modeling, the starting IgG4 model was constructed from crystal structures for Fab B72.3 (PDB: 1BBJ) (36) and serum-derived Fc IgG4 (PDB: 4C55) (44). These have the same sequence as the IgG4 under study. The glycans at Asn297 (Fig. 1 *A*) were retained. The composition of the first glycan was Gal_1_.GlcNAc_2_.Man_3_.GlcNAc_2_; that for the second was a Man_3_.GlcNAc_2_.Fuc core with GlcNAc and Gal.GlcNAc branches. The 20-residue hinge ^216^ESKYGPPCPSCPAPEFLGGP^238^ and the missing C-terminal Fc residues ^442^SLGK^445^ were modeled using the PyMOL build_seq script. All the disulphide bonds within the crystal structures were retained. To complete this structure, force field parameterizations were generated and hydrogen atoms added, as for IgG1 above. Because neither the Cys226-Cys226 or Cys229-Cys229 hinge residues were positioned to form disulphide bonds in the initial structure, these bonds were not included in the force-field-parameterized model of IgG4. The initial model was energy-minimized as for IgG1.

### Configurational sampling of IgG

The hinge conformations of the new starting IgG1 and IgG4 models were rapidly sampled using dihedral Monte Carlo simulations within SASSIE (22) while holding the above energy-minimized Fab and Fc regions fixed. The assigned variable hinge regions of the IgG1 and IgG4 models are listed below for each simulation, and the backbone dihedral angles in these regions were varied. A Metropolis sampling methodology was used to sample the energetically allowed dihedral angles, using only the dihedral component of the CHARMM potential (39) to determine the energy of each configuration. Sterically overlapping IgG structures were automatically discarded in SASSIE.

For IgG1, three Monte Carlo simulations were performed to maximize the sampling of possible conformers with plausible hinge disulphide bonding. Of a total of 704,000 randomized conformations that were generated, 231,492 structural models were sterically acceptable (i.e., no atomic overlaps). The three simulations gave 27,158 models that contained both canonical disulphide bonds as follows:

Simulation 1: 100,000 models were generated, of which 68,914 (68.9%) were sterically acceptable. The lower hinge retained the Cys226-Cys226 disulphide bond, whereas the Cys229-Cys229 bond was not present. The upper-hinge peptide ^220^CDKTHT^225^ was varied in both heavy chains. The asymmetry of the starting crystal structure was retained by this simulation.
Simulation 2: 200,000 models were generated, of which 135,742 (67.9%) were sterically acceptable. Here, the full hinge ^215^VEPKSCDKTHTCPPCPAPELLGGP^238^ was varied. This simulation sampled conformations with and without symmetry but resulted in a low sampling rate for conformers that showed viable disulphide bonding conformations.
Simulation 3: 404,000 models were generated, of which 26,836 (6.6%) were sterically acceptable. The full hinge was again varied, but with the additional constraint that the Cys226-Cys226 and Cys229-Cys229 *α*-carbon atoms had to be within 0.75 nm of each other to permit interchain disulphide bonding. This additional constraint removed many models.

For IgG4, two Monte Carlo simulations generated 700,000 IgG4 randomized conformations, from which 190,437 structural models were sterically acceptable. Simulation 1 for IgG1 was not performed for IgG4 because the Fab and Fc crystal structures used for the starting model did not contain a disulphide-bridged hinge structure. The simulations resulted in 46,979 models that contained both canonical disulphide bonds:

Simulation 2: As for IgG1, 300,000 trial models were generated by varying the full hinge ^215^VESKYGPPCPSCPAPEFLGGP^238^, of which 143,568 models (47.9%) were sterically acceptable. Only 110 models contained both canonical disulphide bonds.
Simulation 3: As for IgG1, 400,000 trial models were generated in which the Cys226-Cys226 and Cys229-Cys229 pairs involved in interchain disulphide bonding in IgG4 had to be within 0.75 nm of one another. From this, 46,869 models (11.7%) were acceptable. As for IgG1, this disulphide bridge constraint removed many models.

### Scattering curve calculations and analyses

Scattering curves for the acceptable IgG1 and IgG4 models were calculated using SCT (21). SCT is a coarse-grained method that converts the atomistic models into small sphere models for the Debye calculation of the theoretical scattering curves *I*(*Q*) (45). For comparison with the SANS data in heavy water buffer, the sphere models were left unhydrated. For comparison with SAXS data, hydration spheres were added to create a monolayer hydration shell corresponding to 0.3 g of water per gram of protein (18,20). The coordinate conversion to spheres used a grid with cube side lengths of 0.5329 nm for IgG1 and 0.5335 nm for IgG4, plus a cutoff of four atoms. SCT optimized these parameters to reproduce the unhydrated protein volume. For comparison with the scattering curves, each experimental *I*(*Q*) value was matched to the theoretical *I*(*Q*) value with the closest *Q* value. For the x-ray curves with up to 365 data points, the *Q* spacing is close enough for this procedure to have little effect, whereas the neutron curves have up to 45 data points and the quality of the matches is reduced (16). After this, the *R* factor was computed by analogy with crystallography in which the lower *R* factors represent better fits:

(4) $$\;R-factor\;=\;\frac{\sum \left|I_{Expt}\left(Q\right)\;-\;\eta I_{Theor}\left(Q\right)\right|}{\sum \left|I_{Expt}\left(Q\right)\right|}\times 100.$$

*η* is a scaling factor used to match the theoretical curve to the experimental *I*(0) value. An iterative search to minimize the *R* factor was used to determine *η*. All steps were performed in the SASSIE-web workflow (version 0.8) (https://sassie-web.chem.utk.edu/sassie2). Structures with the lowest *R* factors were accepted as valid models of the antibody solution structure. We note that *χ*^2^ values are often used elsewhere as a monitor of best fits to scattering curves; these, however, require errors for the experimental intensities that were not always available.

The final antibody structures were analyzed using the distance between the centers of mass of the two Fab regions Fab1 and Fab2 (d1) and those between each Fab region with the Fc region (d2, d3) (Fig. 1 *B*). The twofold symmetry of the antibody primary structure meant that the differentiation between Fab1 and Fab2, and consequently d2 and d3, is only there for descriptive clarity. These parameters were used previously for IgG4 (46) and other antibodies (14,47,48). Antibody asymmetry is monitored by the absolute difference between the two Fab-Fc distances, abs(d2 − d3), which is close to zero for symmetric structures.

### Docking analyses for C1q heads and the Fc*γ*R receptors

Crystal structures for C1q and the Fc*γ*RI receptor were docked onto the best-fit structures for the IgG1 *β* and the IgG4 *α* and *β* clusters using the web server algorithm PatchDock as described previously (15,16). For this, the crystal structure of the C1q head and its predicted contacts was used (49,50), together with the crystal structure for the Fc*γ*RI receptor-Fc complex (PDB: 4X4M (50)). Crystal structures for the Fc*γ*RIII receptor-Fc complex were also used (PDB: 1E4K and 1T89 (51,52)).

## Results

### Experimental scattering curves for IgG1 and IgG4

To determine the atomistic solution structures of human IgG1 and IgG4, reliable scattering curves were required. Data sets from five scattering instruments were obtained for each of the two monoclonal IgG1 6a and IgG4 B72.3 antibodies in both H_2_O and ^2^H_2_O buffers (Table 1). This tested the reproducibility of the scattering curves to be used for modeling. Two x-ray and neutron data sets for each of IgG1 6a and IgG4 B72.3 on instruments ID02 and SANS2d were reused from our previous study (15,16). For IgG1 6a, two new neutron data sets on instruments D11 and D22 were obtained. For IgG4 B72.3, one new x-ray data set from instrument BM29 and one new neutron data set from instrument D22 were obtained. For IgG1 19a, an x-ray data set from instrument ID02 was reused (16), together with two new neutron data sets from instruments D11 and D22. Analytical ultracentrifugation showed that IgG1 and IgG4 were unaffected by protein aggregation but showed minor reversible dimerization (15,16). The minor dimerization observed only for IgG4 in ^2^H_2_O buffer meant that the neutron curves from SANS2d were extrapolated to zero concentration before modeling this (Table 1).

**Table 1:** Structural Parameters for Human IgG1 and IgG4 from X-Ray and Neutron Scattering

Antibody	Experiment	Concentration (mg/mL)	Buffer	*R*_*g*_ (nm)	*R*_*xs1*_ (nm)	*R*_*xs2*_ (nm)
IgG1 6a	x-ray (ID02)	4.00^a^^,^^b^	PBS-137, H_2_O^c^	5.20 ± 0.06	2.61 ± 0.02	1.42 ± 0.04
	neutron (SANS2d)	4.00	PBS-137, ^2^H_2_O^c^	5.18 ± 0.02	2.45 ± 0.01	1.21 ± 0.01
	neutron (SANS2d)	3.00^b^	PBS-137, ^2^H_2_O	5.16 ± 0.05	2.42 ± 0.04	1.25 ± 0.03
	neutron (D11)	3.91	Hepes-137, ^2^H_2_O	5.20 ± 0.04	2.50 ± 0.01	1.21 ± 0.01
	neutron (D22)	0.96	PBS-137, ^2^H_2_O	5.10 ± 0.06	2.61 ± 0.03	1.48 ± 0.04
IgG1 19a	x-ray (ID02)	1.40^a^	PBS-137, H_2_O^c^	5.13 ± 0.03	2.61 ± 0.07	1.50 ± 0.04
	neutron (D11)	2.83	Hepes-137, ^2^H_2_O	5.16 ± 0.04	2.59 ± 0.01	1.25 ± 0.02
	Neutron (D22)	0.96	PBS-137, ^2^H_2_O	5.03 ± 0.06	2.46 ± 0.03	1.32 ± 0.03
IgG4 B72.3	x-ray (ID02)	5.79	PBS-137, H_2_O^d^	5.04 ± 0.05	2.51 ± 0.02	1.37 ± 0.03
	x-ray (BM29)	1.00	PBS-137, H_2_O	5.00 ± 0.02	2.46 ± 0.01	1.36 ± 0.01
	neutron (SANS2d)	0.00 (extrapolated)	PBS-137, ^2^H_2_O^d^	4.77	2.49	1.19
	neutron (D22)	1.00	PBS-137, ^2^H_2_O	5.00 ± 0.05	2.29 ± 0.01	1.09 ± 0.02

The *R*_*g*_, *R*_*xs1*_, and *R*_*xs2*_ fits were performed using the *Q* ranges specified in Materials and Methods.

a Fig. S2*A*.

b The reported Guinier fit corresponds to the neutron curve in Fig. S2*B*.

c Data from Table 1 in (16).

d Data from Table 1 in (15).

The IgG solution structures were parameterized using linear Guinier fits to determine the *R*_*g*_, *R*_*xs*1_, and *R*_*xs*2_ values using the *Q* ranges specified in Materials and Methods. The *Q* ranges for the *R*_*g*_ and *R*_*xs*_ fits were the same as in our previous studies (15,16) to permit the direct comparison of the *R*_*xs*1_ and *R*_*xs*2_ values. The previous values were similar to the mean values from new concentration series for IgG1 and IgG4 from instruments BM29, D11, and D22 (Table 1). For IgG1 6a, the *R*_*g*_ values were 5.10–5.20 nm (Table 1). The *R*_*xs*1_ and *R*_*xs*2_ values occurred in ranges of 2.42–2.61 and 1.21–1.48 nm, respectively. The mostly lower *R*_*xs*1_ and *R*_*xs*2_ values obtained with the neutron data relative to the x-ray values were attributed to the nonvisibility of the hydration shell by neutron scattering in ^2^H_2_O buffer, unlike with x-ray scattering, in which this shell is visible. For IgG1 19a (not used further in this study), the corresponding previous and new *R*_*g*_, *R*_*xs*1_, and *R*_*xs*2_ values were similar to those of IgG1 6a (Table 1), indicating that both showed similar structures. For IgG4 B72.3, the four sets of x-ray and neutron *R*_*g*_ values were similar to each other but were lower compared to IgG1 at 4.77–5.04 nm. The x-ray *R*_*xs*1_ values were 2.46–2.51 nm, which were reduced to 2.29–2.49 nm for the neutron *R*_*xs*1_ values; the x-ray *R*_*xs*2_ values of 1.36–1.37 nm were reduced to 1.09–1.19 nm for the neutron values. These comparisons indicated that the distinct IgG1 and IgG4 solution structures were reproducibly observed on the different instruments.

**Figure 2:**
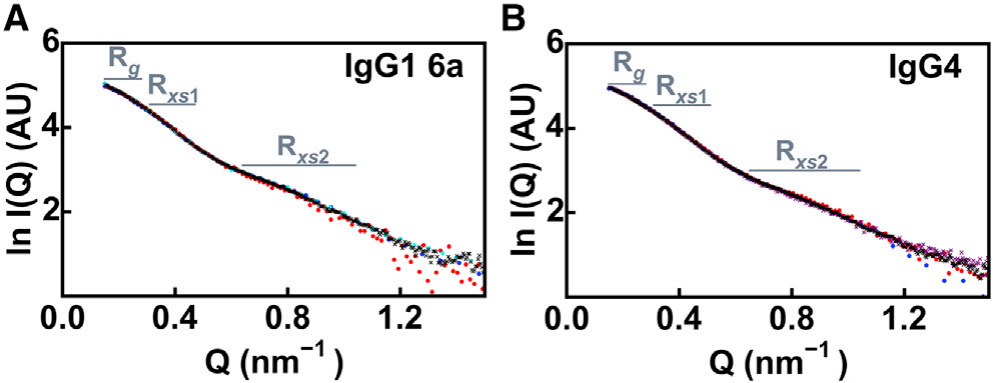
Experimental scattering curves used for the modeling fits. The horizontal bars indicate the *Q* ranges used to calculate the Guinier *R*_*g*_, *R*_*xs1*_, and *R*_*xs2*_ values. The resulting linear Guinier plots are available for inspection in Fig. 6 of (16) for IgG1 and in Fig. S4 of (15) for IgG4. (*A*) For IgG1 6a, the x-ray scattering curve from ID02 (*black crosses*) is compared with the neutron scattering curves from SANS2d (*blue dots*), D11 (*cyan dots*), and D22 (*red dots*). Flat baseline corrections of 2.09 and 0.29% of *I*(0) were subtracted from the *I*(*Q*) curves to allow for different incoherent scattering contributions in the SANS2d and D11 data, respectively, to achieve correspondence with the x-ray data at high *Q*. No correction for incoherent scattering was made for the D22 data. (*B*) For IgG4, the x-ray scattering curves from ID02 (*black crosses*) and BM29 (*purple crosses*) are compared with neutron data from SANS2d (*blue dots*) and D22 (*red dots*). A flat baseline correction of 1.06% of *I*(0) was subtracted from the *I*(*Q*) curves for the SANS2d data to allow for incoherent scattering. No correction was made for the D22 data. To see this figure in color, go online.

For comparison, the full scattering curves out to *Q* = 1.5 nm^−1^ were superimposed on each other based on the forward scattering at zero angle *I*(0) (Fig. 2). The scattering curves for each of IgG1 and IgG4 showed good agreement up to 1.1 nm^−1^. For IgG1, a minor difference was noticed between the SANS2d and D11 neutron curves in the *R*_*xs*2_ fit range of 0.7–1.1 nm^−1^ when these were referenced to the ID02 x-ray curve as baseline (Fig. S1). This difference was attributed to the different effect of the hydration shell on the x-ray and neutron data. Beyond *Q* of 1.1 nm^−1^, the curves generally showed weaker signal/noise ratios; thus, the atomistic scattering modeling fits in this study were only made to a maximal *Q* value of 1.1 nm^−1^ when calculating the goodness-of-fit *R* factors.

### Monte Carlo atomistic modeling of human IgG1

The atomistic solution structural modeling of IgG1 was initiated using the crystal structure of full-length IgG1 b12 (35). Missing amino-acid residues were rebuilt, and the intact IgG1 structure with glycans was energy-minimized by molecular dynamics (Materials and Methods). Next, Monte Carlo simulations were performed with this starting structure, based on three types of conformational variations of the hinges between the Fab and Fc regions (Materials and Methods). Of the generated 704,000 structures, those showing steric overlap were rejected to leave 231,492 physically realistic trial structures for human IgG1. Theoretical scattering curves were then calculated from each model for comparison with experimental data.

**Figure 3:**
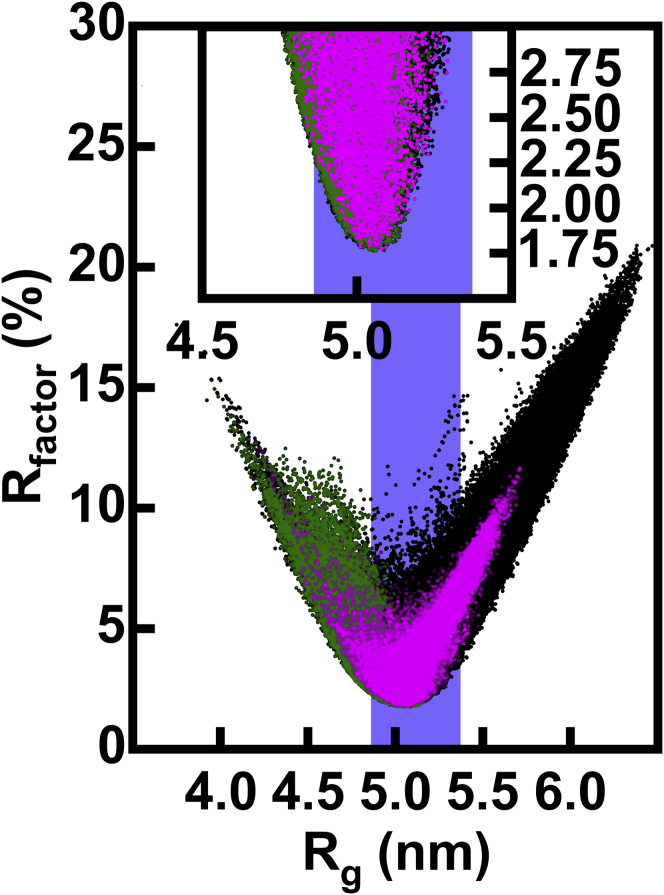
Atomistic modeling analyses of the x-ray scattering curves for IgG1 6a. The 231,492 goodness-of-fit *R* factors for the theoretical *I*(*Q*) curves calculated from the sterically acceptable models of IgG1 are compared to their modeled *R*_*g*_ values, each represented by a single dot. The vertical blue band indicates the modeled *R*_*g*_ values within 5% of the experimental *R*_*g*_ value of 5.20 nm for IgG1 6a (Table 1). Black dots denote all of the 231,492 models. The green dots denote the 68,914 models that retained the Cys226-Cys226 disulphide bridge (Simulation 1). The magenta dots denote the 27,158 models from the simulations in which both the Cys226-Cys226 and Cys229-Cys229 residue pairs were within 0.75 nm of one another to enable disulphide bond formation (26,836 from Simulation 3 and 322 from Simulation 2). The insets show expanded views of the fits for which the *R* factors were below 3.0%. To see this figure in color, go online.

A goodness-of-fit *R* factor analysis was used to identify the errors in the experimental data sets and the *R* factor filter required to select best-fit models. This *R* factor monitored the agreement between the theoretical and experimental curves to select modeled solution structures that were consistent with the experimental curves. To achieve this selection, it was necessary to determine a cutoff *R* factor below which models were assigned as best fits depending on the experimental scattering curve, its signal/noise ratio, and its *Q* range. To determine this cutoff, two experimental curves for the same protein from the same instrument were used to calculate two *R* factors for each of the 231,492 modeled IgG1 curves. The correlation between the two *R* factors was assessed using both the Pearson *r* and Spearman *r*_s_ coefficients (53). By gradually excluding the models with higher *R* factors, this identified the point at which the ranking of the fits was no longer consistently determined for the two experimental curves. The cutoff was chosen as the point where both the *r* and *r*_s_ coefficients decreased below 0.5. It should be noted that this approach is only valid when the best *R* factors were more densely sampled compared to the poor fits, as seen in Fig. 3. Because no detectable difference was seen between the ID02 SAXS curves for IgG1 6a and IgG1 19a (Fig. S2 *A*; Table 1), these two data sets were used to determine the *R* factor cutoff. The same procedure was followed for each of the D11 and D22 SANS curves for IgG1 6a and IgG1 19a (Table 1). Because no SANS2d data were available for IgG1 19a, the SANS2d data for IgG1 6a at 4.0 and 3.0 mg mL^−1^ (Table 1) were compared (Fig. S2 *B*). The final *R* factor cutoffs for IgG1 were determined to be 3.00, 2.00, 3.15, and 3.10% for instruments ID02, SANS2d, D11, and D22, respectively.

First, the IgG1 6a SAXS curve fits based on 231,492 hydrated IgG1 models were performed (Table 2). The three Monte Carlo simulations produced a broad range of conformations with *R*_*g*_ values between 3.75 and 6.47 nm (*black*, Fig. 3). As desired, most of the models occurred near the *R* factor minimum and within error of the experimental *R*_*g*_ value, except for Simulation 3, which incorporated the hinge disulphide constraints. Simulation 1, which utilized the lower hinge conformation from the crystal structure with only one disulphide bond, showed a noticeable sampling bias toward lower *R*_*g*_ values (*green*, Fig. 3). Using the above *R* factor cutoff of 3.00% as the filter (*inset*, Fig. 3), 37,412 (16%) of the 231,492 IgG1 models were deemed consistent with the SAXS curve (Table 2). The 37,412 models were made up of 21,462, 11,291 and 4659 models from Simulations 1, 2, and 3, respectively. Application of a second filter of 0.75 nm for the *α*-carbon separation in the Cys226-Cys226 and Cys229-Cys229 disulphides caused these numbers to fall to 4728 (Table 2), this being distributed as 0, 69, and 4659 models for Simulations 1, 2, and 3, respectively (*magenta*, Fig. 3). The double constraint of *R* factors and disulphide bridges demonstrated the utility of atomistic representations for determining scattering fits. Thus, the requirement of disulphide bridge formation in the models removed many potential structures with low *R* factors.

**Table 2:** Summary of the IgG1 6a Modeling Searches

		X-Ray				Neutron				Structure		
Filter	Number of Models	*R*_*g*_ (nm)	*R*_*xs1*_ (nm)	*R*_*xs2*_ (nm)	*R* factor (%)	*R*_*g*_ (nm)	*R*_*xs1*_ (nm)	*R*_*xs2*_ (nm)	*R* factor (%)	d1 (nm)	max(d2, d3) (nm)	min(d2, d3) (nm)
Experiment (ID02/SANS2d)	n.a.	5.20 ± 0.06	2.61 ± 0.02	1.42 ± 0.04	n.a.	5.18 ± 0.02	2.45 ± 0.01	1.21 ± 0.01	n.a.	n.a.	n.a.	n.a.

**All Models**												

None	231,492	5.21 ± 0.35	2.63 ± 0.27	1.41 ± 0.19	6.0 ± 3.3	4.96 ± 0.28	2.42 ± 0.19	1.35 ± 0.14	3.5 ± 1.6	8.84 ± 1.92	8.50 ± 0.84	7.12 ± 1.04
X-ray *R* factor ≤3.00%	37,412	5.00 ± 0.09	2.63 ± 0.08	1.37 ± 0.09	2.5 ± 0.3	4.79 ± 0.07	2.41 ± 0.06	1.34 ± 0.06	3.3 ± 0.6	8.18 ± 1.45	8.25 ± 0.75	6.78 ± 0.81
X-ray *R* factor ≤3.00% and two disulphides	4728	5.03 ± 0.09	2.63 ± 0.07	1.33 ± 0.08	2.5 ± 0.3	4.82 ± 0.07	2.41 ± 0.05	1.30 ± 0.06	3.1 ± 0.6	9.09 ± 1.08	7.91 ± 0.60	6.51 ± 0.59
X-ray *R* factor ≤3.00%, two disulphides, and neutron *R* factor ≤2.00%	28	5.19 ± 0.04	2.67 ± 0.02	1.33 ± 0.05	2.9 ± 0.1	4.95 ± 0.02	2.45 ± 0.02	1.28 ± 0.03	2.0 ± 0.1	9.68 ± 0.45	8.31 ± 0.53	6.53 ± 0.25

**Cluster *α***												

None	38,388	4.92 ± 0.25	2.64 ± 0.16	1.30 ± 0.23	4.4 ± 1.9	4.72 ± 0.20	2.42 ± 0.13	1.29 ± 0.17	4.1 ± 1.8	6.14 ± 0.67	8.69 ± 0.78	7.58 ± 0.98
X-ray *R* factor ≤3.00%	9016	4.98 ± 0.09	2.63 ± 0.07	1.37 ± 0.08	2.6 ± 0.3	4.78 ± 0.07	2.41 ± 0.06	1.33 ± 0.07	3.4 ± 0.6	6.09 ± 0.51	8.95 ± 0.52	7.87 ± 0.59
X-ray *R* factor ≤3.00% and two disulphides	389	4.97 ± 0.08	2.68 ± 0.06	1.36 ± 0.05	2.6 ± 0.3	4.77 ± 0.06	2.44 ± 0.04	1.31 ± 0.04	3.2 ± 0.5	6.29 ± 0.40	8.77 ± 0.38	7.83 ± 0.49
X-ray *R* factor ≤3.00%, two disulphides, and neutron *R* factor ≤2.00%	0	n.a	n.a	n.a	n.a	n.a	n.a	n.a	n.a	n.a	n.a	n.a

**Cluster *β***												

None	193,104	5.26 ± 0.34	2.63 ± 0.29	1.43 ± 0.17	6.3 ± 3.4	5.00 ± 0.27	2.42 ± 0.20	1.36 ± 0.13	3.3 ± 1.5	9.38 ± 1.60	8.46 ± 0.85	7.03 ± 1.03
X-ray *R* factor ≤3.00%	28,396	5.00 ± 0.09	2.63 ± 0.08	1.37 ± 0.09	2.5 ± 0.3	4.80 ± 0.07	2.41 ± 0.06	1.34 ± 0.06	3.2 ± 0.6	8.84 ± 0.93	8.03 ± 0.67	6.44 ± 0.51
X-ray *R* factor ≤3.00% and two disulphides	4339	5.03 ± 0.08	2.63 ± 0.07	1.32 ± 0.08	2.5 ± 0.3	4.82 ± 0.07	2.41 ± 0.05	1.30 ± 0.06	3.1 ± 0.6	9.34 ± 0.71	7.83 ± 0.55	6.39 ± 0.43
X-ray *R* factor ≤3.00%, two disulphides, and neutron *R* factor ≤2.00%	28	5.19 ± 0.04	2.67 ± 0.02	1.33 ± 0.05	2.9 ± 0.1	4.95 ± 0.02	2.45 ± 0.02	1.28 ± 0.03	2.0 ± 0.1	9.68 ± 0.45	8.31 ± 0.53	6.53 ± 0.25

n.a., not applicable. The first row depicts the experimental values. The three classes of models are defined (see text) and correspond to all the models and their separation into their *α* and *β* clusters. Columns 3–6 refer to the x-ray modeling fits, columns 7–10 refer to the neutron modeling fits, and columns 11–13 specify the modeled inter-Fab and Fc distances defined in Fig. 1*B*.

**Figure 4:**
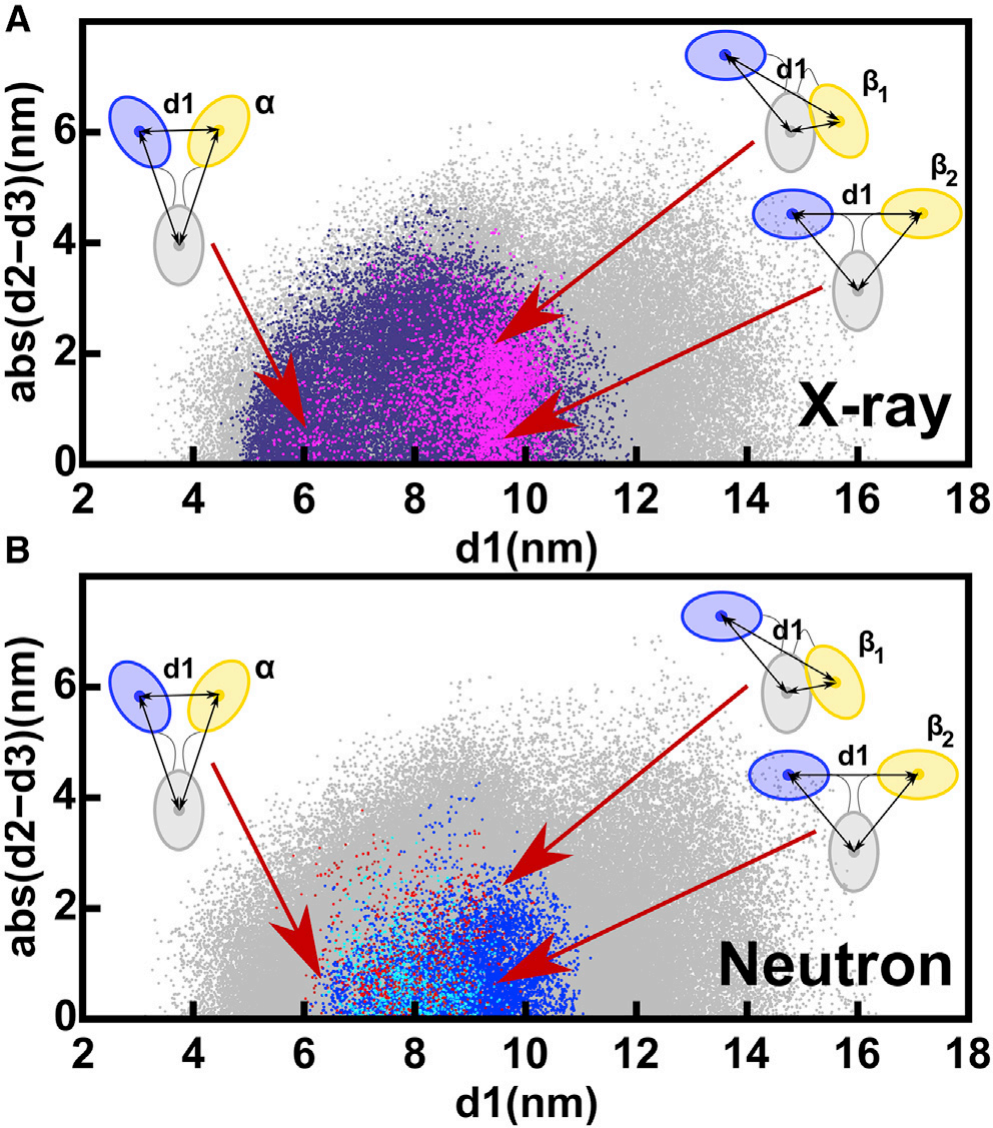
Relationship between the inter-Fab distance (d1) and the absolute difference in Fab to Fc distances, abs(d2 − d3), in the sterically acceptable IgG1 6a x-ray and neutron models. The gray dots represent the 231,492 unfiltered models that represent all the sampled IgG1 conformations (Table 2). (*A*) X-ray modeling of ID02 data. Purple dots represent the 37,412 models with x-ray *R* factors below 3.0% (*top*, Table 2). Of these, the magenta dots represent the better 4728 models in which the Cys226-Cys226 and Cys229-Cys229 residue pairs were both within 0.75 nm of one another (*top*, Table 2). Two clusters of structures *α* and *β* were observed. The *α* cluster at d1 = 6 nm and low abs(d2 − d3) contains 389 symmetric Fab to Fc distances indicated in the cartoon (*middle*, Table 2). The *β* cluster at d1 = 9.5 nm shows 4339 Fab to Fc distances with either asymmetry *β*_1_ or symmetry *β*_2_ (*bottom*, Table 2). (*B*) Neutron modeling. The 10,121, 3121, and 10,836 structures with neutron *R* factors below 2.0% and with both Cys pairs within 0.75 nm of one another are shown (*blue dots*, SANS2d; *red dots*, D11; *cyan dots*, D22) (not shown in Table 2). The *α* and *β* clusters are arrowed as in (*A*). To see this figure in color, go online.

The structural outcome of the IgG1 6a SAXS curve fits was monitored by the d1, d2, and d3 distances between the centers of mass of the Fab and Fc regions (Fig. 1 *B*; (14, 15, 16,46)). Asymmetry was monitored using the absolute difference between the two Fab-Fc distances, abs(d2 − d3). In the 231,492 IgG1 models, the Fab separation d1 ranged between 3 and 16.5 nm (Fig. 4 *A*). Filtering for the above-determined *R* factor for instrument ID02 of 3.00% to give 37,412 models reduced d1 to 4.5–12 nm (*purple*, Fig. 4 *A*). The disulphide distance constraint separated the resulting 4728 models into two clusters (*magenta*, Fig. 4 *A*). The 389 models with d1 of ∼6 nm and below 7 nm were denoted as Cluster *α*. These were Y-shaped symmetric structures because abs(d2 − d3) was low at ∼1 nm. The 4339 models with d1 of ∼9.5 nm and above 7 nm were denoted as Cluster *β*. Those Cluster *β* structures with high asymmetry and a large abs(d2 − d3) were labeled as *β*_1_, whereas those Cluster *β* structures showing symmetry with a low abs(d2 − d3) were labeled as *β*_2_ (*cartoons*, Fig. 4 *A*). These *α* or *β* clusters were only visible through the abs(d2 − d3) difference, not otherwise (Fig. S3, *A* and *B*), hence showing the utility of the abs(d2 − d3) values to evaluate the filtered models.

**Figure 5:**
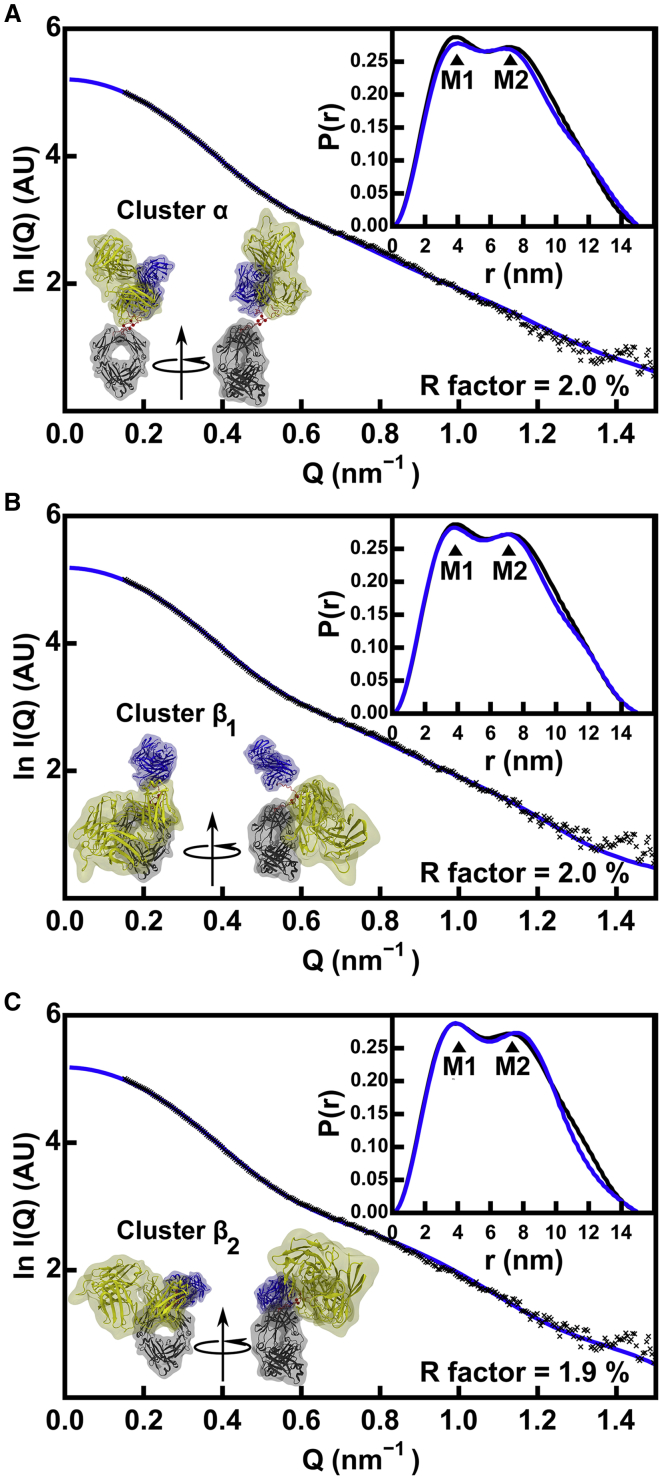
Representative x-ray scattering curve fits for the three families of best-fit IgG1 6a models. The three curve fits correspond to the *α*, *β*_1_, and *β*_2_ clusters (*magenta dots* in Fig. 4*A*). The experimental curve from instrument ID02 is shown in black (Table 1), and the best-fit theoretical curve is shown in blue. The experimental and theoretical distance distribution functions *P*(*r*) are shown at the top right of each panel. For each cluster, the best-fit conformer is shown in two views related by an axial rotation of 90° as indicated to follow the colors of Fig. 1*B*. (*A*) Cluster *α* with a symmetric structure (small abs(d2 − d3)) and a small d1; (*B*) Cluster *β*_1_ with asymmetric Fab-Fc distances (large abs(d2 − d3)) and a large d1; and (*C*) Cluster *β*_2_ with symmetric Fab-Fc distances (small abs(d2 − d3)) and a large d1. To see this figure in color, go online.

The curve fits of the experimental IgG1 SAXS data to the modeled *I*(*Q*) curves calculated from representative Cluster *α*, *β*_1_, and *β*_2_ best-fit structures with the lowest *R* factors revealed very good visual fits (Fig. 5). These *I*(*Q*) fits were corroborated by very good visual fits with the distance distribution function *P*(*r*) for all three Clusters *α*, *β*_1_, and *β*_2_, especially the relative intensities of the M1 and M2 peaks (*insets*, Fig. 5). The final sets of modeled Guinier parameters *R*_*g*_, *R*_*xs*1_, and *R*_*xs*2_ of 4.97 ± 0.08, 2.68 ± 0.06, and 1.36 ± 0.05 nm (Cluster *α*: Table 2) and 5.19 ± 0.04, 2.67 ± 0.02, and 1.33 ± 0.05 nm (Cluster *β*: Table 2) showed a slightly improved agreement of Cluster *β* with the experimental values of 5.20 ± 0.06, 2.61 ± 0.02, and 1.42 ± 0.04 nm (Table 1). Hence, Cluster *β* was more representative of the IgG1 solution structure than Cluster *α*.

For the SANS fits for IgG1 6a, the theoretical curves from the unhydrated 231,492 models were compared with the experimental SANS curves from instruments SANS2d, D11, and D22. The three neutron *R* factor versus *R*_*g*_ graph results (Fig. S4) were similar to those for the SAXS graphs (Fig. 3). In particular, the positions of the *R* factor minima were close to the experimental *R*_*g*_ values. Models with *R* factor values below the cutoffs of 2.00, 3.15, and 3.10%, respectively, were accepted (see above). The models that passed both the *R* factor and disulphide distance filters showed a Fab-Fab separation d1 between 6 and 11 nm (Fig. 4 *B*). The D11 and D22 fits (*red*, *cyan*, Fig. 4 *B*) showed slightly reduced d1 values compared to the SANS2d fits (*blue*, Fig. 4 *B*). Unlike the SAXS modeling, no distinct *α* and *β* clusters were identified. Visual inspection of the curve fits of the theoretical scattering curves with the SANS experimental data for representative structures confirmed that both clusters provide plausible models (Fig. S5). Because the most frequently occurring best SANS-fitted models were consistent with Cluster *β* and showed better agreement with the Guinier parameters *R*_*g*_, *R*_*xs*1_, and *R*_*xs*2_ (Tables 1 and 2), Cluster *β* was concluded to be more representative of the IgG1 solution structure determined by SANS.

### Monte Carlo atomistic modeling of human IgG4

The atomistic modeling of IgG4 was based on two crystal structures for the separate Fab and Fc regions (36,44). To create the starting IgG4 structure, the hinge peptide joining the Fab and Fc regions and four C-terminal residues were modeled, then the intact IgG4 structure with glycans was energy-minimized using molecular dynamics (Materials and Methods). After this, two Monte Carlo simulations were performed. Either the two disulphide bridges in the hinge were disregarded (Simulation 2) or they were present (Simulation 3). Simulation 1 (see above) was not performed because there was no analog of the corresponding IgG1 simulation. A total of 190,437 physically realistic trial structures for human IgG4 were accepted after rejecting the models that showed steric overlap in 700,000 Monte Carlo-generated conformations. As for IgG1, theoretical scattering curves were then calculated from each IgG4 model for comparison with experimental data.

**Figure 6:**
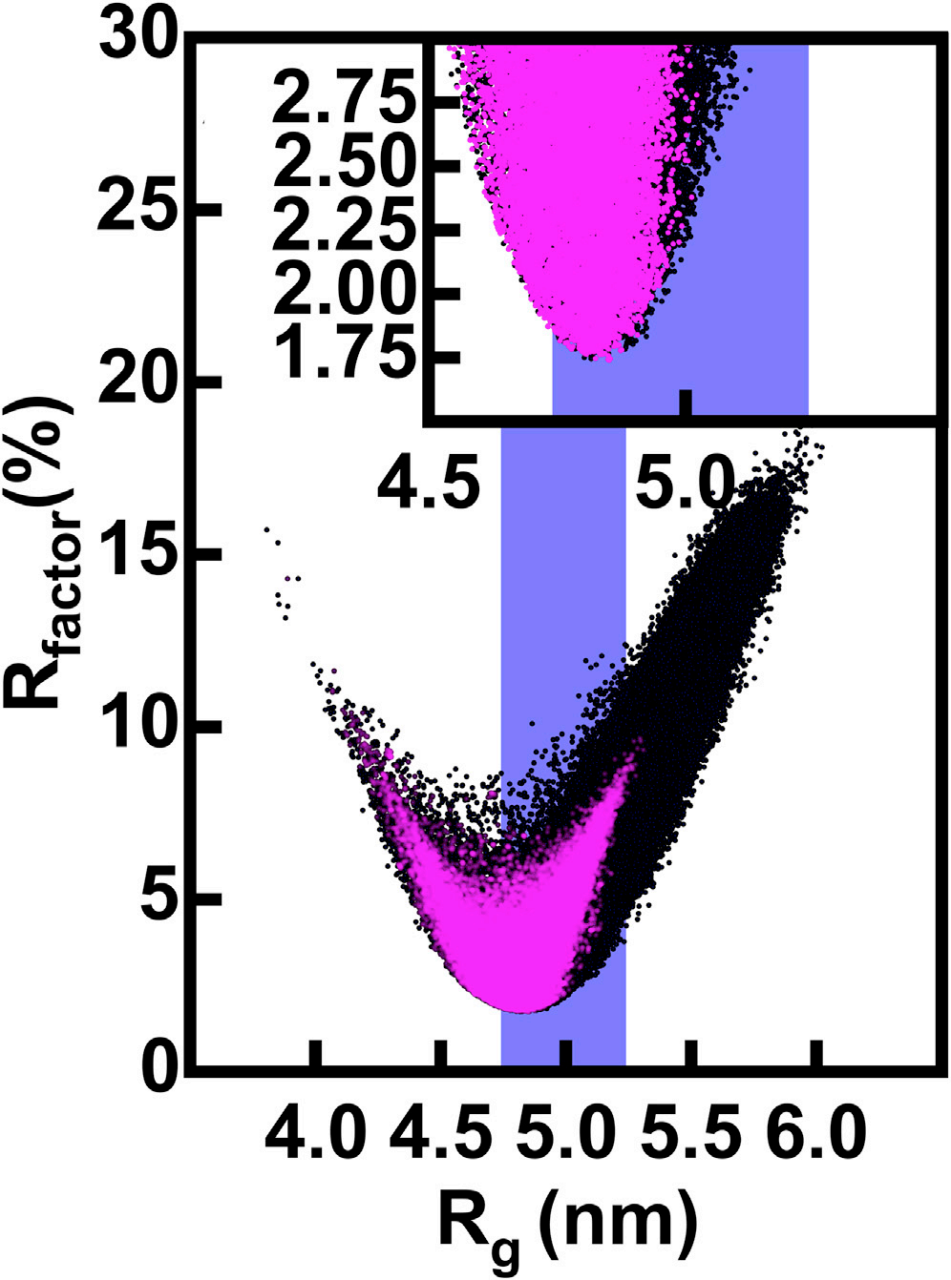
Atomistic modeling analyses of the x-ray scattering curves for IgG4 B72.3. The 190,437 *R* factors for the *I*(*Q*) curves calculated from the sterically acceptable models of IgG4 are compared to their modeled *R*_*g*_ values (Table 3), each being represented by a single dot. The vertical blue shaded band indicates *R*_*g*_ values within 5% of the experimental *R*_*g*_ value of 4.99 nm for IgG4 B72.3 (Table 3). Black denotes all 190,437 models (*top*, Table 3). The magenta overlay denotes the 172,823 models from Simulations 2 and 3 in which the Cys226-Cys226 and Cys229-Cys229 residue pairs are each within 0.75 nm of one another to enable disulphide bond formation. The insets show expanded views of the fits for which the *R* factors were below 3.0%. To see this figure in color, go online.

The 190,437 hydrated IgG4 model structures were analyzed for their fits to the SAXS curves. These gave theoretical curve *R*_*g*_ values that ranged from 3.81 to 6.02 nm (*black*, Fig. 6 *A*). This range was smaller than that of 3.75–6.47 nm sampled for IgG1 (*black*, Fig. 3), and concurred with the lower experimental *R*_*g*_ value of 4.99 ± 0.02 nm for IgG4 (Table 3) compared to that of 5.20 ± 0.06 nm for IgG1 (Table 2). The slightly more compact structure for IgG4 than IgG1 was explained in molecular terms by the IgG4 hinge being three residues shorter than the IgG1 hinge. The majority of the IgG4 sampling was concentrated close to its experimental *R*_*g*_ (Fig. 6). Even though many curves with acceptable *R* factors showed calculated *R*_*g*_ values over 5% lower than the experimental *R*_*g*_ values (*magenta*, Fig. 6), the majority of the good-fit structures including conformations with good disulphide separations of 0.75 nm at the Cys226 and Cys229 pairs have *R*_*g*_ values closer to experiment. Using the two experimental curves (Table 1), an *R* factor cutoff of 3.00% (see above) was determined to give 28,084 models (an acceptance rate of 15%; Table 3). This total was comprised of 13,189 models (9% accepted) from the unconstrained Simulation 2 and 14,895 models (32% accepted) from the disulphide-constrained models of Simulation 3. Filtering for those models on the basis of *R* factors and disulphide separations reduced the 28,084 models to 14,927 models (8% accepted), with 32 (0.02% accepted) and 14,895 (32% accepted) for Simulations 2 and 3, respectively (Table 3). The correct disulphide connectivity thus improved the fits for the IgG4 solution structure.

**Table 3:** Summary of the IgG4 Modeling Searches

		X-Ray				Neutron				Structure		
Filter	Number of Models	*R*_*g*_ (nm)	*R*_*xs1*_ (nm)	*R*_*xs2*_ (nm)	*R* factor (%)	*R*_*g*_ (nm)	*R*_*xs1*_ (nm)	*R*_*xs2*_ (nm)	*R* factor (%)	d1 (nm)	max(d2, d3) (nm)	min(d2, d3) (nm)
Experiment (ID02/SANS2d)	n.a.	4.99 ± 0.02	2.50 ± 0.03	1.37 ± 0.02	n.a.	4.77 ± 0.04	2.49 ± 0.03	1.37 ± 0.03	n.a.	n.a.	n.a.	n.a.

**All Models**												

None	190,437	5.07 ± 0.29	2.66 ± 0.19	1.30 ± 0.22	6.4 ± 3.4	4.84 ± 0.24	2.43 ± 0.15	1.24 ± 0.17	3.8 ± 1.3	7.99 ± 1.78	8.43 ± 0.64	7.26 ± 0.98
X-ray *R* factor ≤3.00%	28,084	4.82 ± 0.09	2.56 ± 0.07	1.36 ± 0.12	2.5 ± 0.3	4.63 ± 0.07	2.35 ± 0.06	1.32 ± 0.10	3.3 ± 0.5	6.82 ± 1.50	8.38 ± 0.65	6.95 ± 1.07
X-ray *R* factor ≤3.00% and two disulphides	14,927	4.80 ± 0.08	2.57 ± 0.07	1.35 ± 0.12	2.5 ± 0.3	4.62 ± 0.07	2.36 ± 0.06	1.31 ± 0.09	3.3 ± 0.5	6.78 ± 1.24	8.45 ± 0.56	6.90 ± 1.02
X-ray *R* factor ≤3.00%, two disulphides, and neutron *R* factor ≤2.85%	2748	4.87 ± 0.06	2.64 ± 0.03	1.25 ± 0.07	2.6 ± 0.3	4.68 ± 0.05	2.42 ± 0.02	1.22 ± 0.05	2.7 ± 0.1	6.80 ± 1.10	8.64 ± 0.43	7.04 ± 0.95

**Cluster *α***												

None	59,668	4.87 ± 0.20	2.62 ± 0.15	1.29 ± 0.25	4.1 ± 1.6	4.67 ± 0.16	2.39 ± 0.13	1.26 ± 0.20	3.3 ± 0.9	5.98 ± 0.71	8.64 ± 0.53	7.69 ± 0.83
X-ray *R* factor ≤3.00%	16,900	4.82 ± 0.09	2.56 ± 0.07	1.37 ± 0.13	2.5 ± 0.3	4.63 ± 0.07	2.35 ± 0.06	1.33 ± 0.10	3.3 ± 0.5	5.74 ± 0.52	8.68 ± 0.40	7.68 ± 0.66
X-ray *R* factor ≤3.00% and two disulphides	8663	4.82 ± 0.08	2.56 ± 0.07	1.35 ± 0.13	2.5 ± 0.3	4.63 ± 0.06	2.35 ± 0.06	1.31 ± 0.10	3.3 ± 0.5	5.82 ± 0.54	8.69 ± 0.37	7.62 ± 0.65
X-ray *R* factor ≤3.00%, two disulphides, and neutron *R* factor ≤2.85%	1645	4.88 ± 0.06	2.63 ± 0.03	1.25 ± 0.07	2.6 ± 0.3	4.69 ± 0.05	2.42 ± 0.02	1.22 ± 0.05	2.7 ± 0.1	5.98 ± 0.39	8.80 ± 0.29	7.70 ± 0.59

**Cluster *β***												

None	130,769	5.16 ± 0.28	2.68 ± 0.20	1.31 ± 0.20	7.5 ± 3.4	4.92 ± 0.22	2.45 ± 0.15	1.23 ± 0.15	4.0 ± 1.3	8.91 ± 1.30	8.33 ± 0.67	7.06 ± 0.97
X-ray *R* factor ≤3.00%	11,184	4.81 ± 0.10	2.56 ± 0.08	1.34 ± 0.12	2.5 ± 0.3	4.63 ± 0.08	2.36 ± 0.06	1.30 ± 0.09	3.3 ± 0.5	8.45 ± 0.91	7.94 ± 0.71	5.85 ± 0.45
X-ray *R* factor ≤3.00% and two disulphides	6264	4.78 ± 0.08	2.58 ± 0.07	1.34 ± 0.11	2.5 ± 0.3	4.61 ± 0.06	2.37 ± 0.05	1.30 ± 0.09	3.3 ± 0.5	8.09 ± 0.53	8.10 ± 0.59	5.91 ± 0.44
X-ray *R* factor ≤3.00%, two disulphides, and neutron *R* factor ≤2.85%	1103	4.85 ± 0.05	2.64 ± 0.03	1.25 ± 0.06	2.6 ± 0.3	4.67 ± 0.04	2.43 ± 0.02	1.22 ± 0.05	2.7 ± 0.1	8.04 ± 0.48	8.41 ± 0.48	6.04 ± 0.29

n.a., not applicable. The first row depicts the experimental values. The three classes of models are defined (see text) and correspond to all the models and their separation into their *α* and *β* clusters. Columns 3–6 refer to the x-ray modeling fits, columns 7–10 refer to the neutron modeling fits, and columns 11–13 specify the modeled inter-Fab and Fc distances defined in Fig. 1*B*.

**Figure 7:**
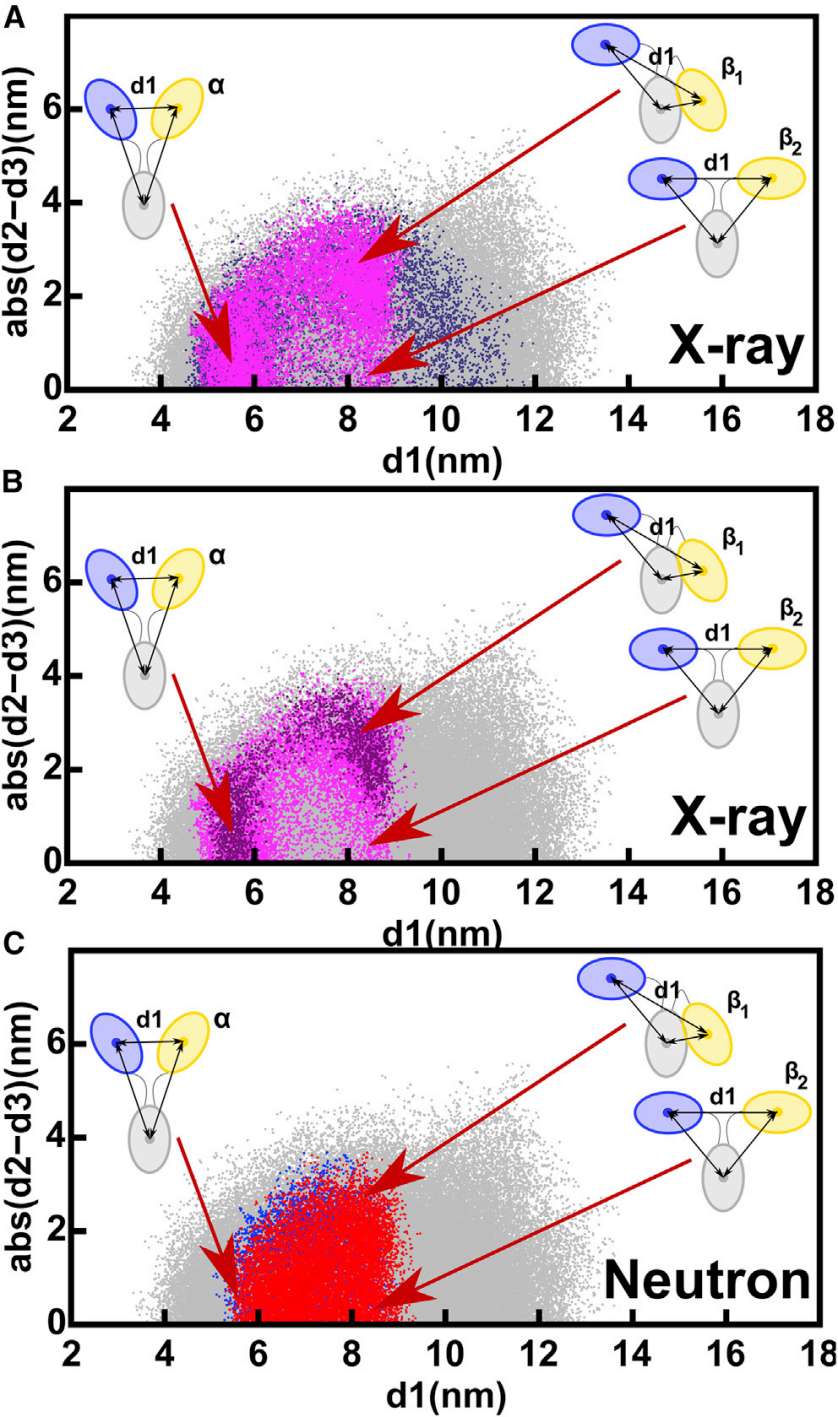
Relationship between the inter-Fab distance, d1, and the absolute difference in Fab to Fc distances, abs(d2 − d3) in the IgG4 x-ray and neutron models. In all subpanels, gray dots represent the 190,437 unfiltered models that represent all the sampled IgG4 conformations. (*A*) X-ray modeling of ID02 data. Purple dots represent the 28,084 models in which the *R* factor is less than 3.0% (*top*, Table 3). Magenta dots represent the better 14,927 models with *R* factors below 3.0% in which the Cys226-Cys226 and Cys229-Cys229 residue pairs were both within 0.75 nm of one another. As for IgG1 (Fig. 4*A*), two groups of structures were observed. The *α* cluster at d1 = 5.5 nm and low abs(d2 − d3) values contains 8663 symmetric Fab-to-Fc distances, indicated in the cartoon (*middle*, Table 3). The *β* cluster at d1 between 8 and 8.5 nm shows 6264 distances with high asymmetry (large abs(d2 − d3) values) and low asymmetry (small abs(d2 − d3) values) labeled *β*_1_ and *β*_2_, respectively, indicated in the cartoons (*bottom*, Table 3). (*B*) X-ray modeling of BM29 data. The same filtered modeling results from the BM29 data in a darker hue (3647 models) are compared with the ID02 data in magenta (14,927 models; (*A*)). The *α* and *β* clusters are arrowed as in (*A*). (*C*) Neutron modeling. The 45,975 and 66,092 structures that provided acceptable *R* factors below 2.85% for the neutron fits are shown (*blue dots*, SANS2d; *red dots*, D22). In these structures, both Cys pairs were within 0.75 nm of one another. The *α* and *β* clusters are arrowed as in (*A*). To see this figure in color, go online.

The structural outcome of the IgG4 SAXS curve fits was monitored by the distances between the centers of mass of the Fab and Fc regions, as for IgG1 above. The 190,437 models showed a Fab separation d1 that extended to 14 nm (*gray*, Fig. 7 *A*). Filtering for *R* factors below 3.00% limited d1 to a range between 5 and 12 nm (*purple*, Fig. 7 *A*). Interestingly, the maximum d1 value of 12 nm was similar to that for IgG1 (*purple*, Fig. 4 *A*). Application of the hinge disulphide constraint resulted in the observation of two clusters, *α* and *β*, that corresponded to d1 being below or above 7 nm, respectively (*magenta*, Fig. 7 *A*). Cluster *β* in IgG4 showed a smaller average d1 of 8.09 nm compared to 9.34 nm in IgG1 (Tables 2 and 3). Unlike IgG1, the models in Cluster *β* were predominantly asymmetric, with abs(d2 − d3) values mostly found at 2.5 nm and labeled *β*_1_. Cluster *β*_2_ was much less populated for IgG4 compared to IgG1. When the IgG4 structures were filtered using curve fits based on the SAXS instrument BM29 with an *R* factor cutoff of 2.40%, very similar Clusters *α*, *β*_1_, and *β*_2_ were again visible (*dark magenta*, Fig. 7 *B*), thus confirming the reproducibility of the modeling fits from two different SAXS data sets.

**Figure 8:**
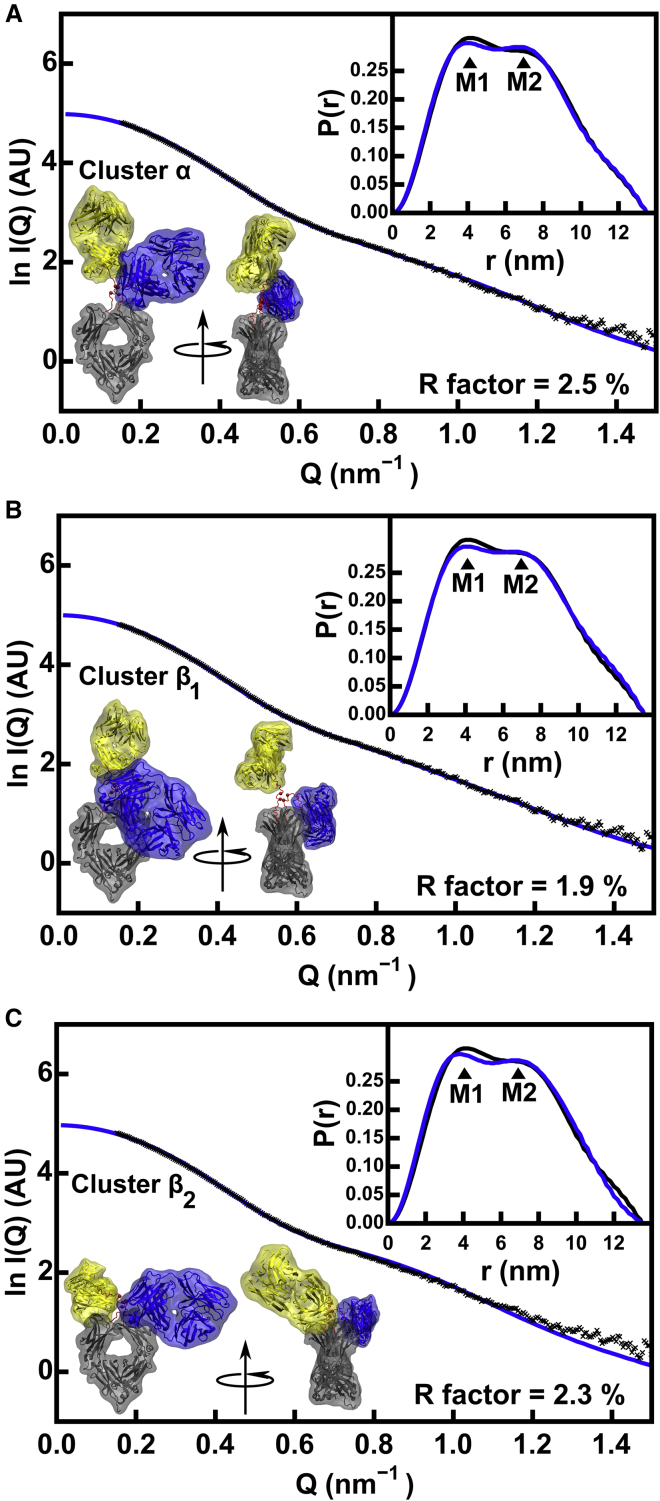
Representative x-ray scattering curve fits for the three families of best-fit IgG4 B72.3 models. The three curve fits correspond to the *α*, *β*_1_, and *β*_2_ clusters of Fig. 7*A* with *R* factors below 3.0% and Cys pairs within 0.75 nm of one another. The experimental curve from instrument ID02 is shown in black (Table 1; (15)), and the best-fit theoretical curve is shown in blue. The experimental and theoretical distance distribution functions *P*(*r*) are shown at the top right of each panel. The best-fit conformer is shown in two views related by an axial rotation of 90° to follow the colors of Fig. 1*B*. (*A*) Cluster *α* with a symmetric structure (small abs(d2 − d3)) and a small d1; (*B*) Cluster *β*_1_ with asymmetric Fab-Fc distances (large abs(d2 − d3)) and a large d1; (*C*) Cluster *β*_2_ with symmetric Fab-Fc distances (small abs(d2 − d3)) and a large d1. To see this figure in color, go online.

Very good visual experimental SAXS fits to the modeled scattering curves *I*(*Q*) were seen for representative best-fit IgG4 structures for Clusters *α*, *β*_1_, and *β*_2_ that had the lowest *R* factors (Fig. 8). These were corroborated by very good visual fits with the distance distribution function *P*(*r*), including the relative intensities of the M1 and M2 peaks in the *P*(*r*) curves (*insets*, Fig. 8). The final modeled Guinier parameters *R*_*g*_, *R*_*xs*1_, and *R*_*xs*2_ of 4.82 ± 0.08, 2.56 ± 0.07, and 1.35 ± 0.13 nm (Cluster *α*) and 4.78 ± 0.08, 2.58 ± 0.07, and 1.34 ± 0.11 nm (Cluster *β*) were in good agreement with the experimental values of 4.99 ± 0.02, 2.50 ± 0.03, and 1.37 ± 0.02 nm, respectively (Tables 1 and 3). Similar *R*_*g*_, *R*_*xs*1_, and *R*_*xs*2_ values were obtained with or without the disulphide distances constraint, showing the importance of the atomistic modeling approach to narrow the number of allowed structures. The mean *R*_*g*_ values of both clusters were similar to the experimental value of 4.99 nm, showing that these clusters could not be distinguished.

For the SANS fits, the theoretical curves for the unhydrated 190,437 IgG4 models were compared with each of the SANS IgG4 curves from instruments SANS2d and D22 (Table 1). Using the two experimental neutron curves (Table 1), the *R* factor cutoff for acceptable models was determined to be 2.85% for both data sets. After filtering for the hinge disulphide separations, the Fab-to-Fab separation d1 for acceptable models was between 5 and 9.5 nm (Fig. 7 *C*; Table 3). No separate *α* and *β* clusters were resolved for IgG4 in the neutron fits, unlike the x-ray fits, although the same range of d1 values was seen. Unlike the SANS modeling for IgG1, which showed fewer models in Cluster *α*, many of the good-fit SANS models for IgG4 occurred in both the *α* and *β* clusters. Visual inspection of curve fits between theoretical scattering curves and the SANS experimental data for representative structures confirm that both clusters provide plausible models (Fig. S6). This means that IgG4 models from both the *α* and *β* clusters were consistent with both the SAXS and SANS data. This outcome was attributed to the shorter hinge in IgG4, which permitted alternative arrangements of the two Fab and one Fc regions in the intact antibody.

### Joint fits of SAXS and SANS curves for IgG1 and IgG4

The SAXS atomistic modeling analyses for IgG1 and IgG4 resulted in 4728 and 14,927 conformational models, respectively (Tables 2 and 3), that satisfied the *R* factor cutoff and disulphide distance constraint (Figs. 4 and 7). These x-ray-modeled structures were based on hydrated proteins with a surface monolayer of water. The SANS-modeled structures differed in that unhydrated proteins, in which the surface monolayer of water molecules was mostly invisible (20), were visualized. The combination of the different views from the x-ray and neutron modeling should narrow the ranges of accepted structures. By using the above-determined *R* factor cutoffs of 3.00% (IgG1, IgG4, ID02), 2.00% (IgG1, SANS2d), and 2.85% (IgG4, SANS2d), far fewer modeled structures satisfied these double x-ray and neutron filters.

**Figure 9:**
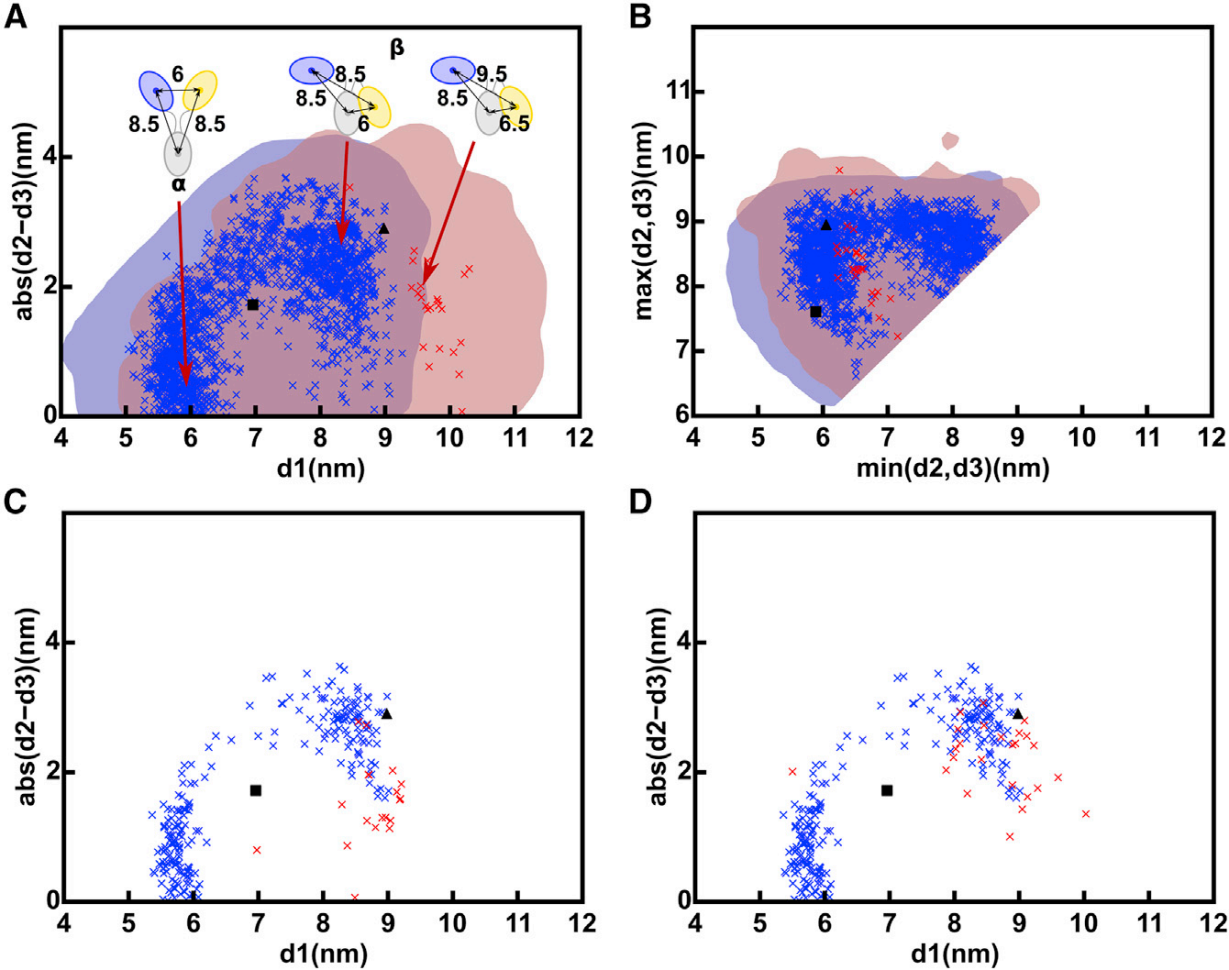
Summary of the joint best-fit x-ray and neutron models and connected hinge disulphide bonds. Their x-ray and neutron *R* factors are below 3.0%, and the two Cys pairs are within 0.75 nm of one another. The red and blue crosses indicate the ensembles of the best-fit IgG1 and IgG4 models respectively. In (*A*) and (*B*), the red and blue shaded areas represent the libraries of 231,492 and 190,437 structures for IgG1 and IgG4, respectively, in the Monte Carlo simulations. In (*A*)–(*D*), ▲ and ■ denote the IgG1 and IgG4 crystal structures, respectively (PDB: 1HZH and 5DK3). (*A*) A comparison of the 28 and 2748 d1 and abs(d2 − d3) distances after the theoretical IgG1 and IgG4 models were filtered, using both the ID02 x-ray and SANS2d neutron data together (Table 4). The cartoons illustrate representative *α* structures and two distinct *β* structures for IgG4 (*left*) and IgG1 (*right*) in this plot, in which the numbers denote the separations in nm. Substantial overlap is seen in the starting Monte Carlo conformations for IgG1 and IgG4 before filtering. (*B*) Distribution of the smaller Fab-Fc (min(d2, d3)) and larger Fab-Fc distances (max(d2, d3)) for each of the 28 and 2748 IgG1 and IgG4 models filtered in (*A*). (*C* and *D*) A comparison with (*A*) of the d1 and abs(d2 − d3) distributions derived from repeat experimental data sets. Using the ID02 x-ray data jointly with the (*C*) D11 neutron data resulted in 24 IgG1 models and with the (*D*) D22 neutron data resulted in 26 IgG1 models, shown in red crosses (Table 4). The 2845 IgG4 models (*blue crosses*) were filtered using the BM29 x-ray data and D22 neutron data in both (*C*) and (*D*) (Table 4). To see this figure in color, go online.

1) For IgG1, the weak evidence above for Cluster *α* conformations was confirmed by the exclusion of Cluster *α* in the double x-ray and neutron fits (*red*, Fig. 9 *A*). There, Cluster *β* was centered at d1 = 9.68 ± 0.45 nm (Table 4). This outcome was attributed to a more extended IgG1 hinge conformation that does not permit the formation of the more compact conformations that correspond to Cluster *α.* This outcome was reproducible when the SANS2d neutron data were replaced by neutron data from D11 (*red*, Fig. 9 *C*) and D22 (*red*, Fig. 9 *D*). There, Cluster *β* was centered at similar d1 values of 9.06 ± 1.06 and 9.08 ± 1.06 nm (Table 4).

**Table 4:** Joint SAXS*-*SANS Fits for the Best-Fit IgG1 and IgG4 Models

Paired Data Source	Cluster	Number of Models	*R*_*g*_ (nm) Mean ± SD	d1 (nm) Mean ± SD	max(d2, d3) (nm) Mean ± SD	min(d2, d3) (nm) Mean ± SD
**IgG1 best-fit models**						

ID02-SANS2d	*β*	28	5.19 ± 0.04	9.68 ± 0.45	8.31 ± 0.53	6.53 ± 0.25
ID02-D11	all^a^	24	5.03 ± 0.08	9.08 ± 1.06	7.89 ± 0.58	6.53 ± 0.56
ID02-D22	all^a^	26	5.03 ± 0.08	9.06 ± 1.06	7.91 ± 0.58	6.58 ± 0.55

**IgG4 best-fit models**						

ID02-SANS2d	all	2748	4.87 ± 0.06	6.80 ± 1.10	8.64 ± 0.43	7.04 ± 0.95
	*α*	1645	4.88 ± 0.06	5.98 ± 0.39	8.80 ± 0.29	7.70 ± 0.59
	*β*	1103	4.85 ± 0.05	8.04 ± 0.48	8.41 ± 0.48	6.04 ± 0.29
BM29-D22	all	2845	4.89 ± 0.04	6.83 ± 1.29	8.76 ± 0.33	7.15 ± 1.14
	*α*	1641	4.90 ± 0.04	5.78 ± 0.24	8.86 ± 0.27	8.04 ± 0.42
	*β*	1204	4.89 ± 0.04	8.34 ± 0.36	8.62 ± 0.35	5.84 ± 0.16

The best-fit structures were filtered on the basis of their simultaneous agreement with the paired x-ray and neutron data sets indicated in each section. All the accepted IgG1 and IgG4 models have the disulphide-bridged Cys226-Cys226 and Cys229-Cys229 residues within 0.75 nm of one another, as required for bonding (Fig. 1*A*). Columns 5–7 specify the modeled inter-Fab and Fc distances defined in Fig. 1*B*. SD, standard deviation.

a The D11 filter retained five Cluster *α* structures, and the D22 filter retained a single Cluster *α* structure (not tabulated).
2) For IgG4, both the Cluster *α* and *β* models passed the double x-ray and neutron filter. Both clusters showed similar populations when tested against the joint ID02-SANS2d data sets (Table 4). An explanation for why IgG4 existed in two alternative good-fit conformations was provided from Fig. 9, *A* and *B* (in *blue*). In Clusters *α* and *β*, two of the three separations d1, d2, and d3 in IgG4 were 6.8–7.0 nm, and the third was 8.6 nm (Table 4), unlike IgG1, for which the three separations were distinct at 9.1, 7.9, and 6.5 nm (Table 4). Given that the Fab and Fc regions were similar in sizes, Clusters *α* and *β* in IgG4 were, in fact, indistinguishable. The same separations for IgG4 were also seen in the joint BM29-D22 data sets (*blue*, Fig. 9, *C* and *D*); thus, this outcome was reproducible.
3) Crystal structures for full-length human IgG1 and IgG4 provided an independent assessment of the atomistic modeling, although each one only provided a single snapshot of one structure. The IgG1 crystal structure (PDB: 1HZH) gave separations d1 = 9.0 nm and d2 and d3 = 9.0 and 6.1 nm that resembled Cluster *β* (35), indicating that the crystal and solution structures were similar (▲, Fig. 9). The IgG4-based pembrolizumab crystal structure (PDB: 5DK3) gave separations d1 = 7.0 nm and d2 and d3 = 7.6 and 5.9 nm that were intermediate between Cluster *α* and *β* (▪, Fig. 9) (47). Consequently, although similar to the solution structure, the IgG4 crystal structure did not distinguish between the two clusters. Although further MD simulations could potentially suggest a preference for either Cluster *α* or *β* conformations, further insight by using this was considered unlikely.

## Discussion

We have described in detail a, to our knowledge, new atomistic method to determine solution structures of full-length human IgG1 and IgG4 antibodies by joint SAXS and SANS studies. After data collection, we constructed a full-sized energy-minimized molecular model for each of IgG1 and IgG4, then submitted these to Monte Carlo simulations at their hinges to generate a large and broad range of structures to enable best-fit structures to be determined. This method opens new avenues for future structural studies of full-length antibodies of all types. The steps in this process are outlined. First, abundant x-ray and neutron scattering data for monoclonal human IgG1 and IgG4 in light and heavy water buffers from five different instruments were used. The joint data sets established their experimental reproducibility, defined the appropriate *R* factors for filtering based on experimental curve comparisons, and provided two different views of hydrated and unhydrated IgG structures. To model these data sets, molecular dynamics first ensured that the starting antibody protein structures for IgG1 and IgG4 based on crystal structures were physically realistic and complete. Each starting structure was then inputted into the SASSIE-web modeling workflow, in which Monte Carlo randomization of the antibody hinge structure was performed (23). The Monte Carlo approach offered a computationally rapid means of generating 700,000 and 704,000 trial structures for full-length IgG1 and IgG4, respectively. After sterically overlapping IgG structures were removed, 231,492 and 190,437 acceptable structures, respectively, were identified (Tables 2 and 3). Filters based on x-ray *R* factors of 2.00–3.15% and a hinge disulphide separation of 0.75 nm reduced these totals to 4728 and 14,927, respectively (Tables 2 and 3). As a third filter, by accepting only those structures that jointly fitted the x-ray and neutron data sets, these structures were further reduced to final totals of 28 and 2748, respectively (Tables 2 and 3). The comparison of these filtered best-fit models with the starting acceptable structures (Figs. 3 and 6) showed that enough structures had been sampled and that convergence to best-fit structures was encompassed within these starting structures. The 28 accepted IgG1 solution structures were asymmetric; these fell into a Cluster *β* group of structures with separations d1 of 9.7 nm and d2 and d3 of 8.3 and 6.5 nm (Fig. 1 *B*; Table 4). For IgG4, two final totals were identified, one being 1645 symmetric structures (Cluster *α*) with separations d1 of 6.0 nm and d2 and d3 = 8.8 and 7.7 nm and the other being 1103 asymmetric structures (Cluster *β*) with separations d1 of 8.0 nm and d2 and d3 = 8.4 and 6.0 nm (Table 4). Other searches based on different joint x-ray and neutron data sets (Table 4) reported similar outcomes. These final 28 and 2748 best-fitted IgG1 and IgG4 structures are downloadable as Data S1.

**Figure 10:**
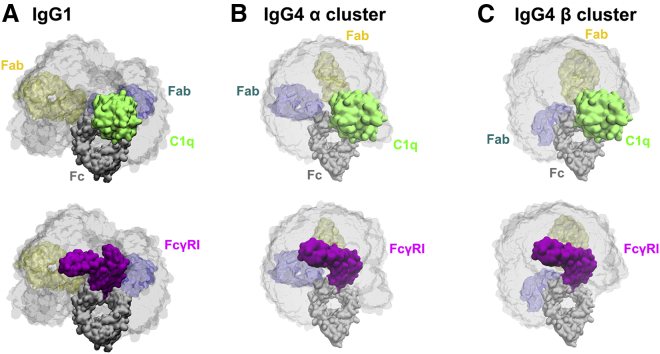
Receptor binding to the best-fit IgG1 (*A*) and IgG4 (*B* and *C*) models. The density plots show the Fc structure in a gray surface representation and the sterically accepted Fab structures in a gray semitransparent volume. Representative best-fit Fab orientations for the IgG1 *β* cluster and the IgG4 *α* and *β* clusters are shown in blue and yellow as labeled. The upper row shows IgG1 or IgG4 bound to a docked C1q head (*green envelope*), and the bottom row shows IgG1 or IgG4 bound to the Fc*γ*RI receptor (*purple*). In all views, the Fc structure is viewed face-on. To see this figure in color, go online.

New, to our knowledge, biological insights were obtained from these atomistic IgG1 and IgG4 solution structures. First, by considering the hinge peptides as molecular structures in the fits, we have an atomistic explanation for the more elongated solution structure of IgG1 compared to that of IgG4. The atomistic modeling also showed that the IgG1 solution structure is asymmetric, this asymmetry being similar to that of the IgG1 crystal structure (Fig. 9 *A*). Second, these molecular structures provided new functional insights on the molecular basis for IgG1 and IgG4 complex formation with two major ligands, i.e., the globular heads of complement C1q and the high-affinity Fc*γ*RI receptor (Fig. 10). Here, atomistic modeling now based on molecular dynamics and Monte Carlo simulations, compared to our less detailed 2014 modeling (15,16), provided a clearer molecular explanation of the relative reactivities of IgG1 and IgG4.

1) For IgG1, the 28 best-fit IgG1 solution structures (Cluster *β*) were combined with a docking model for the interaction between human IgG1 Fc and the crystal structure of the C1q globular head, this being taken from our 2015 study (16,49,50). Overlap was defined as a docked C1q structure at either of the two Fc sites showing >50 heavy atoms within 0.2 nm for either Fab region. Only three (10%) of the 28 IgG1 best-fit structures showed overlap. In the case of the crystal structure of the human Fc-Fc*γ*RI receptor, considered in the same way (16,54), only four (14%) of the 28 IgG1 structures showed overlap. The low degrees of overlap indicated that the C1q-IgG1 and Fc*γ*RI-IgG1 interactions were permitted by the IgG1 solution structures without too much displacement of the Fab regions (Fig. 10 *A*). Similar results were obtained for crystal structures for the Fc-Fc*γ*RIII complexes, showing that the IgG1-Fc*γ*RIII interactions were permitted (51,52).
2) For Cluster *α* in IgG4, the combination of the 1645 best-fit IgG4 structures with the C1q head and the Fc*γ*RI receptor showed 109 (7%) and 1340 (82%) steric overlap of the two ligands with the Fab regions, respectively (Fig. 10 *B*). For Cluster *β* for IgG4, the combination of the 1103 best-fit IgG4 structures with the C1q head and the Fc*γ*RI receptor showed 203 (18%) and 1009 (95%) steric overlap of the two ligands with the Fab regions, respectively (Fig. 10 *C*). The low overlap for the C1q-IgG4 interaction indicated that this interaction was permitted, provided that the shorter IgG4 hinge showed enough flexibility to enable the Fab regions to be displaced for complex formation. The high overlap for the Fc*γ*RI receptor indicated that IgG4 would show reduced reactivity for binding to its receptor, making this interaction unlikely. High overlap was seen also for the Fc*γ*RIII receptor, making this interaction unlikely, too.

Our IgG1 and IgG4 studies complement other recent investigations on antibody solution structures. A neutron spin echo study (55) was conducted with a heterogeneous mixture of polyclonal monomeric and dimeric human IgG from plasma, comprised of the four IgG1, IgG2, IgG3, and IgG4 subclasses with different hinge structures between the subclasses. Similar to earlier studies of antibody flexibility involving the Fab and Fc regions, flexibility was detected as contributions to translational and rotational diffusion motions between the Fab and Fc regions. Interestingly, this flexibility does not influence our averaged structure determinations for the IgG1 and IgG4 subclasses, given that these were able to account for their different receptor-binding functions. Other related studies have examined different aspects of antibodies, for which our approach is relevant. For example, antibodies are important as pharmaceutical proteins, and the study of their aggregation propensities is important to control these for their effectiveness. A multidisciplinary biophysical approach was used to study the unfolding, interactions, and aggregation pathways for a set of four human IgG1 monoclonal antibodies (56). Although no atomistic modeling was performed in that study, there is much scope for modeling to be applied, as illustrated by our recent atomistic study of the unfolding of a Fab region at low pH by a combination of SAXS, molecular dynamics, and FRET analyses (57). A polydisperse mixture of monomeric and dimeric bovine IgG, IgA, and IgM has also been studied, also going to very high concentrations but with examples of bead modeling and fits to the human IgG1 b12 crystal structure that provided some insight into the scattering data (58). Finally, other molecular modeling studies have been reported for antibodies, although less extensive than this study. One study related to the modeling of glycan chains in four small proteins using ab initio bead methods, including the antibody Fc region (59). Another related to the construction of a full human IgG1 structure from its Fab and Fc regions by molecular dynamics. Despite the approximations made in this modeling, this approach successfully accounted for diffusion coefficients measured using dynamic light scattering (60). SASSIE has already been used to study antibodies. A structure for human monoclonal IgG2 was determined from SANS data, although the models were based on a mouse IgG2a crystal structure and not a human IgG2 crystal structure (13). The use of this mouse structure may have affected the outcome of this analysis because the hinges are different between human and mouse IgG2. In a subsequent SAXS and SANS study of human myeloma IgG2, as many as 400,000 trial models were generated for IgG2 over a longer hinge region with the correct hinge sequence (61). This enabled IgG2 function to be assessed. A more ambitious SASSIE modeling analysis was undertaken for human IgG2 binding to a tetrameric streptavidin antigen (62). Experimentally, multiple complexes were formed based on monodentate and bidentate complexes. Nonetheless, the SASSIE modeling showed that compact models for the bidentate antibody-antigen complexes fitted well with the SAXS data.

The atomistic modeling of antibody solution structures from scattering data has evolved significantly since its original inception in 1995. Our first approach used systematic translations and rotations of separate Fab and Fc crystal structures in bovine IgG to fit SANS data, followed by the molecular modeling of the two hinge peptides into the best-fit Fab-Fc arrangement (63). Our second strategy utilized molecular dynamics to generate conformationally randomized hinge peptides to connect the Fab and Fc regions in human IgA1 antibody, thereby creating intact randomized structures for scattering fits (64). This strategy gave 14 different antibody best-fit solution scattering structures that were deposited in the PDB (21). Drawbacks included the lengthy processing of trial structures and an inefficient exclusion of sterically overlapping Fab and Fc regions. Our current (and third) strategy in SASSIE started from an energy-minimized structure, used rapid Monte Carlo sampling methods on a high-performance computing platform, rejected structures with poor stereochemistry at the point of generation, and employed an integrated modeling workflow for scattering fits (22). In this way, human IgG2 antibody was modeled from SANS data, and human IgA1 antibody likewise from SAXS and SANS data sets (13,65). This third approach will enable more ambitious modeling of antibody solution structures in the future, whether this will be to determine new types of structures or to monitor conformational changes.

These IgG1 and IgG4 atomistic structural fits yield significantly more information from scattering experiments than previously. For this, molecular dynamics and Monte Carlo methods proved indispensable. Even though the conformational difference between IgG1 and IgG4 was evident from the x-ray and neutron scattering data (Table 1), the atomistic modeling that directly fitted these scattering data enabled a molecular structural interpretation of this difference (Fig. 10). Interestingly, the 28 asymmetric best-fit structures for IgG1 6a resembled the IgG1 crystal structure, thus showing reproducibility and consistency with other structural methods (Fig. 9). Also interestingly, the crystal structure for the IgG4-based therapeutic antibody pembrolizumab showed consistency with the 2748 symmetric and asymmetric best-fit structures, although the crystal structure was intermediate between the Cluster *α* and *β* solution structures. Alongside these scattering fits, the availability of high-quality scattering curves was indispensable. Here, experimental errors in these curves were considered by comparing side-by-side two independent experimental scattering curves of the same protein, then computing the *R* factor to show the extent of agreement between these. Our curve comparison of data from two different x-ray and three different neutron instruments showed that the lowest *R* factors ranged between 2.00 and 3.15%. The quality of the modeled fits surpassed this experimental limit for both IgG1 and IgG4, with *R* factors below 3% in the full scattering curve *Q* range out to 1.5 nm^−1^ (Tables 2 and 3), indicating that the generation of 700,000 trial structures was sufficient for accurate atomistic modeling. The modeling resulted in an ensemble of related structures (Fig. 9), not one definitive solution structure. The application of three constraints (low *R* factors, disulphide bridge connection, joint x-ray and neutron fits) much reduced the number of allowed structures in the ensemble. In the future, the advent of size-exclusion chromatography in SAXS and SANS data collection will improve the quality of the scattering curves by the removal of trace aggregates, leading to improved modeling outcomes.

Atomistic scattering modeling systematically evaluates all physically allowed conformations once enough models are sampled. This modeling procedure raises the issue of how to interpret the resulting conformations. Previously, principal component analyses were used to identify four distinct clusters of structures that defined the asymmetric solution structure of human IgA1 (65). Here, the alternative representation of the fitted solution structures based on plots of abs(d2 − d3) vs. d1 resulted in two different conformations, termed Clusters *α* and *β*, that gave indistinguishable scattering fits. Cluster *α* featured the two Fab regions ∼6 nm apart and both separated by ∼8.0–8.5 nm from the Fc region in mostly symmetric structures (Table 4). Cluster *β* showed the two Fab region separations and one of the Fab-Fc separations at ∼8.0–8.5 nm in mostly asymmetric structures, with the other Fab-Fc separation at ∼6 nm (Fig. 9 *A*). The fit ambiguity resulted from the similar sizes and shapes of the Fab and Fc regions, meaning that these could be interchanged and still give good fits. For IgG1, this ambiguity was resolved by the disulphide constraint and the use of joint x-ray and neutron fits; this eliminated Cluster *α* to leave Cluster *β* as the best-fit solution structure. For IgG4, its shorter hinge meant that both Clusters *α* and *β* offered good fits, with no clear preference for either conformation. It is possible that the two clusters represent degeneracies in the modeling and not two distinct structural outcomes. Nonetheless, this result still enabled the analysis of IgG4 binding to its C1q and Fc*γ*RI ligands by docking. This outcome emphasizes the importance of combining multiple experimental data sets to understand protein solution structures, especially when combining solution data with crystal and NMR structures (66).

In recent years, several algorithms have been developed to construct solution structure ensembles for experimental SAXS and SANS data (reviewed in (23)). A protein in solution is often considered to exist in multiple structural states with a population distribution, and a trajectory from a molecular dynamics simulation is an example of one such ensemble. Given that the experimental scattering curve is a time-averaged observation of protein structural states in solution, the question arises whether any of the determined protein structures give an experimental scattering curve that is significantly different from the experimental one. This is unlikely for IgG1 and IgG4 because, from the algorithm in use, all the generated structures were stereochemically valid without steric clashes. The Monte Carlo simulations were sufficiently broad that they were able to generate quite different multiple structural states. Nonetheless, each of the IgG1 and IgG4 fits resulted in a single distribution with one clear minimum, implying that there is a single structural type for both proteins (Figs. 3 and 6; Fig. S4). Given that there were no indications of alternative structures, our computational method here and in our earlier studies (15,16) determines the average structure that fits the experimental data. Thus, the molecular dynamics and Monte Carlo modeling approach used here becomes a method that fits one structural ensemble to the experimental data.

## Conclusions

We have reported here in detail a new, to our knowledge, new modeling method to determine solution structures of full-length human IgG1 and IgG4 antibodies by joint SAXS and SANS studies. This opens new avenues for future structural studies of full-length antibodies. New biological insights were obtained on the IgG1 and IgG4 solution structures. The IgG1 and IgG4 atomistic structural fits have yielded significantly more information from scattering experiments than previously.

## Author Contributions

D.W.W. and E.L.K.E. designed and performed the modeling analyses and wrote the manuscript. G.K.H. obtained SAXS and SANS data and analyzed these. S.J.P. conceived and coordinated the study and wrote the manuscript.

## Supporting Material

Document S1. Figs. S1–S6 and Table S1

Data S1. Zip File Containing Coordinates of Final Best-Fit Models

Document S2. Article plus Supporting Material
