# Supplementary material for: Atomistic Modeling of Scattering Curves for Human IgG1/4 Reveals New Structure-Function Insights
Source: Biophys J. 2019 Oct 24;117(11):2101–19. doi: 10.1016/j.bpj.2019.10.024 (PMC6895691; doi:10.1016/j.bpj.2019.10.024)
Supplement: Document S1. Figs. S1–S6 and Table S1 [file mmc1.pdf]

Biophysical Journal, Volume *117*

Supplemental Information

Atomistic Modeling of Scattering Curves for Human IgG1/4 Reveals New Structure-Function Insights

David W. Wright, Emma L.K. Elliston, Gar Kay Hui, and Stephen J. Perkins

SUPPORTING MATERIAL

Atomistic modelling of scattering curves for human IgG1/4 reveals new structure-function insights

David W. Wright, Emma L. K. Elliston, Gar Kay Hui and Stephen J. Perkins

From the Department of Structural and Molecular Biology, Division of Biosciences, Darwin Building, University College London, Gower Street, London WC1E 6BT, U.K.

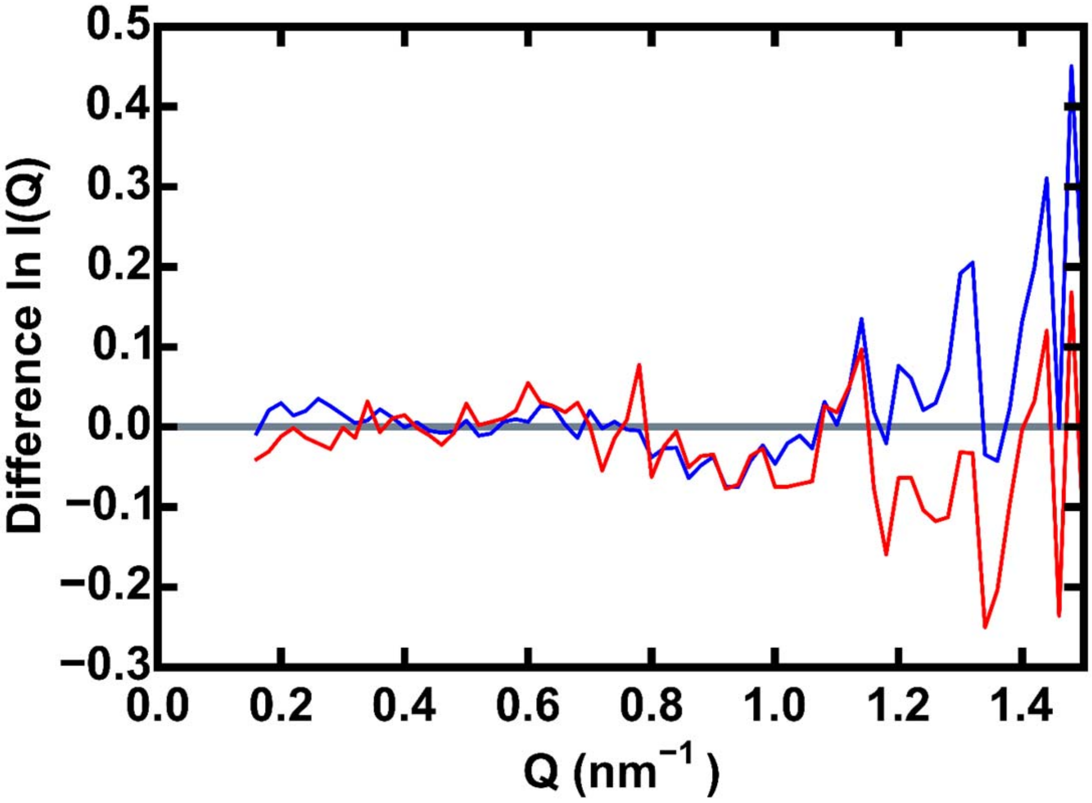

SUPPLEMENTARY FIGURE S1. Difference between the IgG1 6a neutron scattering curves from SANS2D (blue) and D11 (red) and the ID02 X-ray data (grey baseline). The curves were interpolated onto a grid with 0.02 nm^{-1} spacing in *Q*. A dip in both sets of differences was visible between 0.75 and 1.1 nm^{-1} relative to the X-ray baseline, being attributable to the hydration shell.

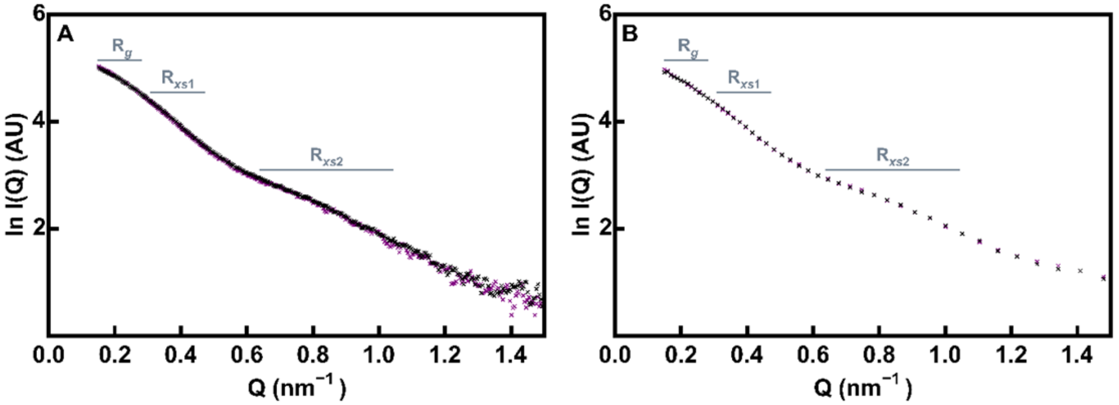

SUPPLEMENTARY FIGURE S2. Curve comparisons used to define the *R* factor cut-off in the IgG1 X-ray and neutron modelling. Experimental details are given in Table 1.

A, The ID02 X-ray data from IgG1 6a (4.0 mg/ml; black) and IgG1 19a (1.9 mg/ml purple) are superimposed.

B, The ID02 X-ray data for IgG1 6a at 4.0 mg/ml (black) is superimposed with the SANS2d neutron data for IgG1 6a at 3.0 mg/ml (purple).

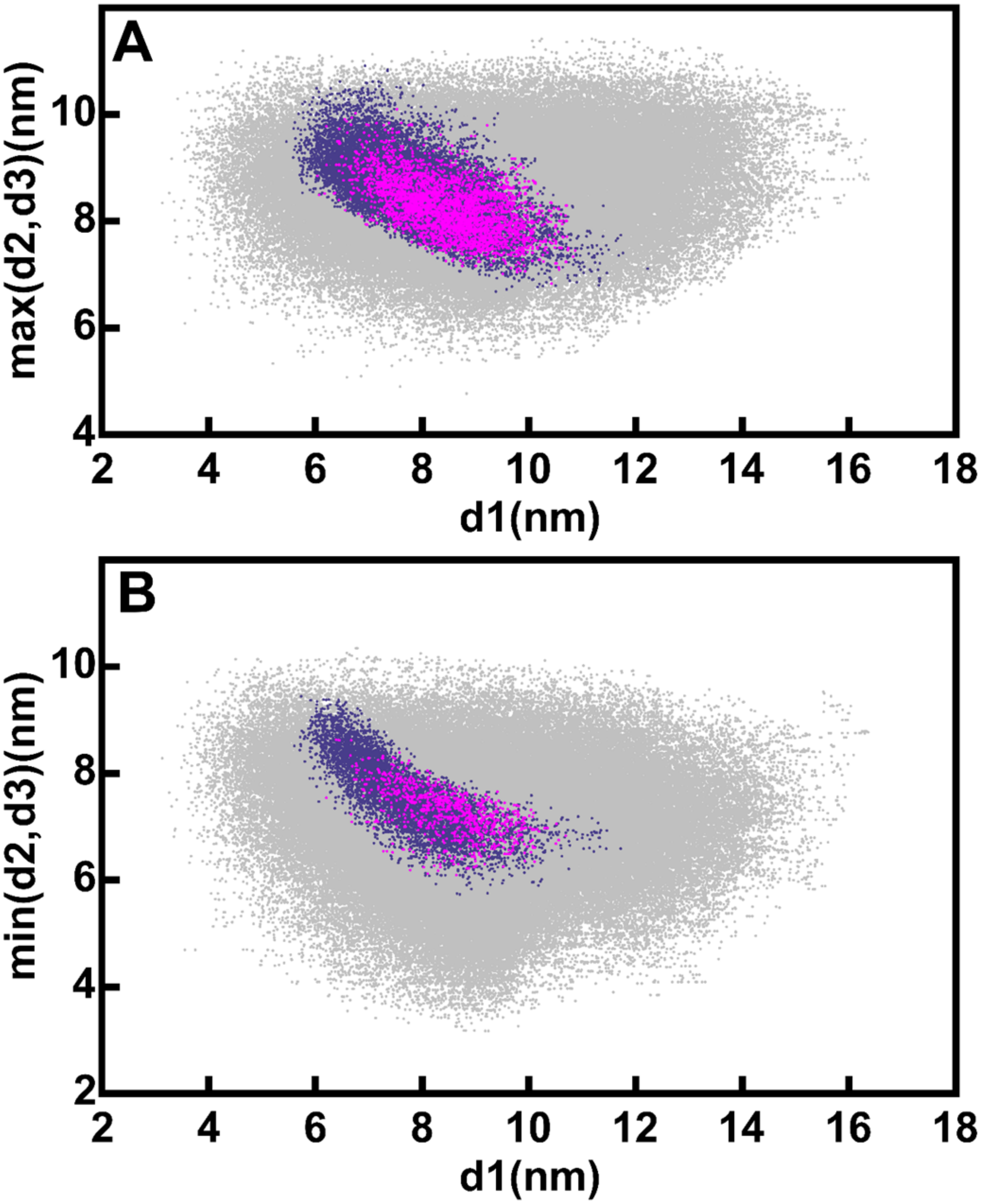

SUPPLEMENTARY FIGURE S3. Distribution of the inter-Fab distance (d1) and the larger and smaller Fab-Fc distances (d2, d3) produced by Monte Carlo simulations of IgG1 6a. The distances d1, d2 and d3 are defined in Fig. 1B. Grey dots represent all 231,492 models that represented the unfiltered sampled conformations (Table 2). Purple dots represent the 37,412 models where the R factors were less than 3.0%. Magenta dots represent the 4,728 models when the R factors were less than 3.0% and additionally the Cys226-Cys226 and Cys229-Cys229 residue pairs were both within 0.75 nm of one another.

A, Larger Fab-Fc distance, max(d2,d3) vs. inter-Fab distance, d1.

B, Smaller Fab-Fc distance, min(d2,d3) vs. inter-Fab distance, d1.

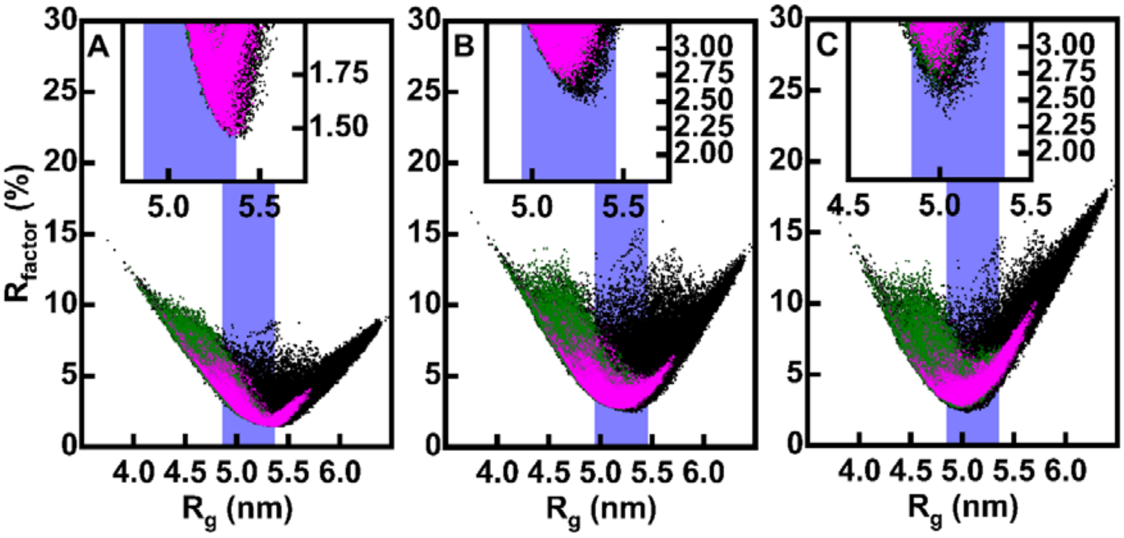

SUPPLEMENTARY FIGURE S4. Three atomistic modelling analyses based on the neutron scattering curves from three instruments for IgG1 6a. This follows the X-ray analyses of Figure 3. The 231,492 goodness-of-fit R factors for the *I(Q)* curves calculated from the sterically acceptable models of IgG1 are compared to their modelled R_g values. The vertical blue regions indicates the modelled R_g values within 5% of the experimental R_g value of 5.20 nm for IgG1 6a (Table 1). Black denotes all 231,492 models. The green overlay denotes the 68,914 models that retained the Cys226-Cys226 disulphide bridge (Simulation 1). The magenta overlay denotes the 27,158 models from the simulations in which both the Cys226-Cys226 and Cys229-Cys229 residue pairs were within 0.75 nm of one another to enable disulphide bond formation (26,836 from Simulation 3, and 322 from Simulation 2). The insets show expanded views of the fits for which the R factors were below 3.0%.

A, For SANS2d, the 231,492 individual models are represented by single dots.

B, For D11, the 231,492 individual models are represented by single dots.

C, For D22, the 231,492 individual models are represented by single dots.

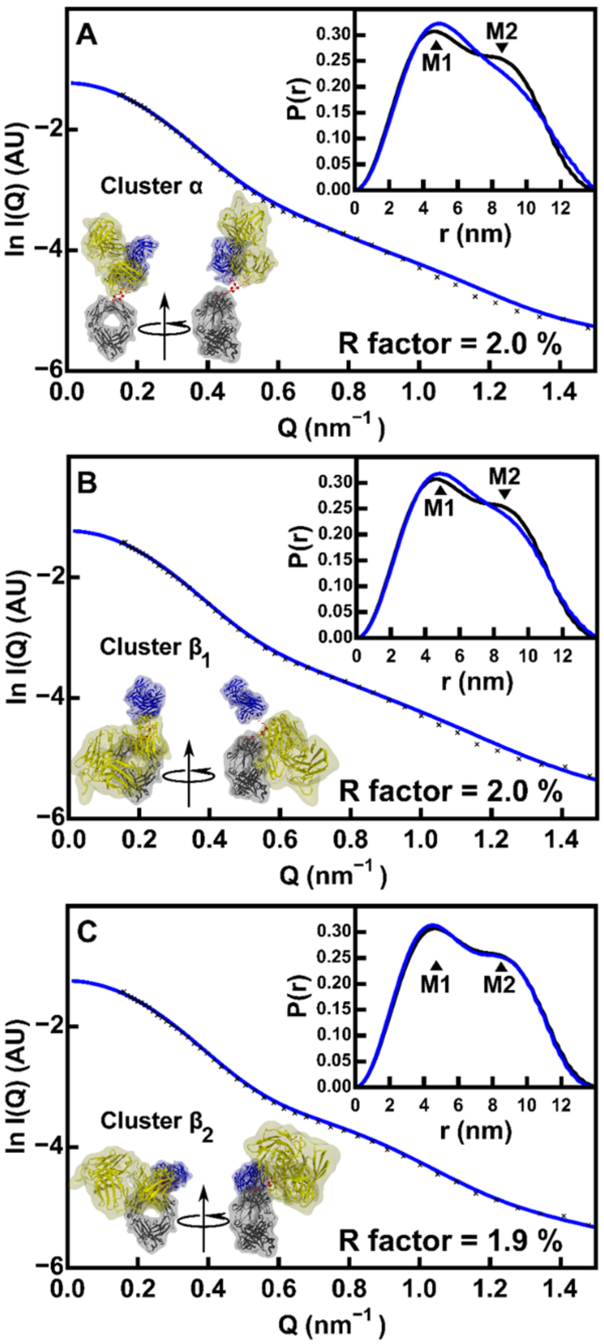

SUPPLEMENTARY FIGURE S5. Representative neutron scattering curve fits for the three families of best-fit IgG1 6a models. The three curve fits correspond to the α, β_1 and β_2 clusters (arrowed in Figure 4B). The experimental curve is shown in black, and the best-fit theoretical curve is shown in blue. Errors are not shown as these are small. The experimental and theoretical distance distribution functions *P(r)* are shown at the top right of each panel. For each cluster, the best-fit conformer is shown in two views related by an axial rotation of 90º as indicated to follow the colours of Figure 1B.

A, cluster α with a symmetric structure (small abs(d2-d3)) and a small d1;

B, cluster β_1 with asymmetric Fab-Fc distances (large abs(d2-d3)) and a large d1;

C, cluster β_2 with symmetric Fab-Fc distances (small abs(d2-d3)) and a large d1.

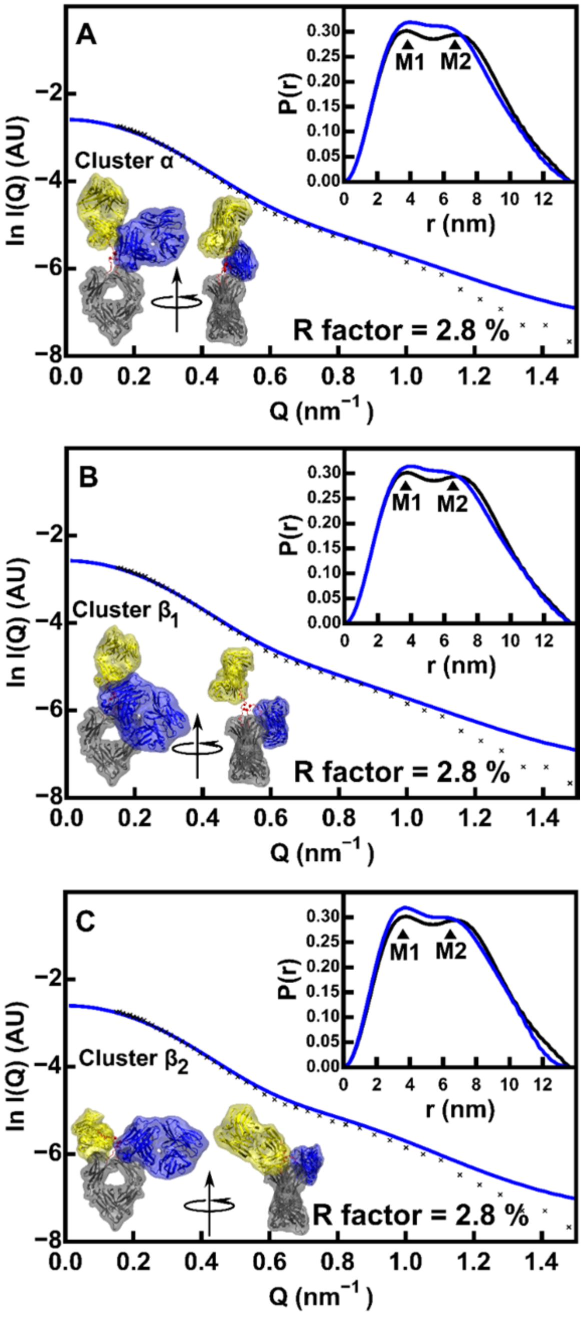

SUPPLEMENTARY FIGURE S6. Representative neutron scattering curve fits for the three families of best-fit IgG4 B72.3 models. The three curve fits correspond to the α, β_1 and β_2 clusters of Figure 7C with *R* factors below 3.0% and Cys pairs within 0.75 nm of one another. The experimental curve is shown in black, and the best-fit theoretical curve is shown in blue. Errors are not shown as these are small. The experimental and theoretical distance distribution functions *P(r)* are shown at the top right of each panel. The best-fit conformer is shown in two views related by an axial rotation of 90º to follow the colours of Figure 1B.

A, cluster α with a symmetric structure (small abs(d2-d3)) and a small d1;

B, cluster β_1 with asymmetric Fab-Fc distances (large abs(d2-d3)) and a large d1;

C, cluster β_2 with symmetric Fab-Fc distances (small abs(d2-d3)) and a large d1.

Supplementary Table S1. Statistics from the modelling searches of IgG1 6a and IgG4 jointly filtered using the X-ray and neutron experimental data and their hinge disulphide connectivity. The X-ray data originate from Instruments ID02 and BM29. The neutron data originate from Instruments SANS2d, D11 and D22. Other terminology is defined in the main text.

**Experiment**	**Filter ***	**Cluster**	**Mean Rg (nm)**	**Mean Rxs1 (nm)**	**Mean Rxs2 (nm)**	**Mean R factor (%)**	**Mean d1 (nm)**	**Mean min(d2,d3) (nm)**	**Mean max(d2, d3) (nm)**
**IgG1**									
ID02/SANS2d	R factor ≤ 3.00 %/2.00 % and two disulphides	all	5.19 ± 0.04	2.67 ± 0.02	1.33 ± 0.05	2.90 ± 0.07	9.68 ± 0.45	8.31 ± 0.53	6.53 ± 0.25
ID02/ D11	R factor ≤ 3.00 %/3.15 % and two disulphides	all	5.03 ± 0.08	2.64 ± 0.07	1.32 ± 0.08	2.49 ± 0.30	9.08 ± 1.06	7.89 ± 0.58	6.53 ± 0.56
ID02/D22	R factor ≤ 3.00 %/2.85% and two disulphides	all	5.19 ± 0.04	2.67 ± 0.02	1.33 ± 0.05	2.90 ± 0.07	9.68 ± 0.45	8.31 ± 0.53	6.53 ± 0.25
**IgG4**									
ID02/SANS2d	R factor ≤ 3.00 %/2.85 % and two disulphides	all	4.89 ± 0.07	2.63 ± 0.04	1.26 ± 0.08	2.57 ± 0.37	6.88 ± 1.42	8.56 ± 0.57	7.11 ± 1.04
ID02/SANS2d	R factor ≤ 3.00 %/2.85 % and two disulphides	α	4.87 ± 0.20	2.62 ± 0.15	1.29 ± 0.25	3.40 ± 1.32	5.98 ± 0.71	8.64 ± 0.53	7.69 ± 0.83
ID02/SANS2d	R factor ≤ 3.00 %/2.85 % and two disulphides	β	4.88 ± 0.06	2.63 ± 0.03	1.25 ± 0.07	2.56 ± 0.40	5.98 ± 0.39	8.80 ± 0.29	7.70 ± 0.59
BM29/D22	R factor ≤ 2.40%/3.10 % and two disulphides	all	4.89 ± 0.04	2.58 ± 0.03	1.32 ± 0.05	2.34 ± 0.14	6.83 ± 1.29	8.76 ± 0.33	7.15 ± 1.14
BM29/D22	R factor ≤ 2.40%/3.10 % and two disulphides	α	4.91 ± 0.04	2.58 ± 0.03	1.34 ± 0.06	2.39 ± 0.16	5.74 ± 0.24	8.89 ± 0.28	8.09 ± 0.42
BM29/D22	R factor ≤ 2.40%/3.10 % and two disulphides	β	5.16 ± 0.28	2.68 ± 0.20	1.31 ± 0.20	4.83 ± 2.23	8.91 ± 1.30	8.33 ± 0.67	7.06 ± 0.97

* The two R factor cut-offs correspond to the two instruments specified in the left column in that order.
